# Opto‐Electrochemical Dissolution Reveals Coccolith Calcium Carbonate Content

**DOI:** 10.1002/anie.202108435

**Published:** 2021-08-15

**Authors:** Minjun Yang, Christopher Batchelor‐McAuley, Samuel Barton, Rosalind E. M. Rickaby, Heather A. Bouman, Richard G. Compton

**Affiliations:** ^1^ Physical and Theoretical Chemistry Laboratory Department of Chemistry University of Oxford South Parks Road Oxford UK; ^2^ Department of Earth Sciences University of Oxford South Parks Road Oxford UK

**Keywords:** analytical methods, calcite dissolution, electrochemistry, global carbonate cycle, marine phytoplankton

## Abstract

Coccoliths are plates of biogenic calcium carbonate secreted by calcifying marine phytoplankton; annually these phytoplankton are responsible for exporting >1 billion tonnes (10^15^ g) of calcite to the deep ocean. Rapid and reliable methods for assessing the degree of calcification are technically challenging because the coccoliths are micron sized and contain picograms (pg) of calcite. Here we pioneer an opto‐eletrochemical acid titration of individual coccoliths which allows 3D reconstruction of each individual coccolith via in situ optical imaging enabling direct inference of the coccolith mass. Coccolith mass ranging from 2 to 400 pg are reported herein, evidencing both inter‐ *and* intra‐species variation over four different species. We foresee this scientific breakthrough, which is independent of knowledge regarding the species and calibration‐free, will allow continuous monitoring and reporting of the degree of coccolith calcification in the changing marine environment.

## Graphical abstract

Acid dissolution enables 3D image reconstruction of coccoliths, providing mass estimation in the range of 2 to 400 picograms.

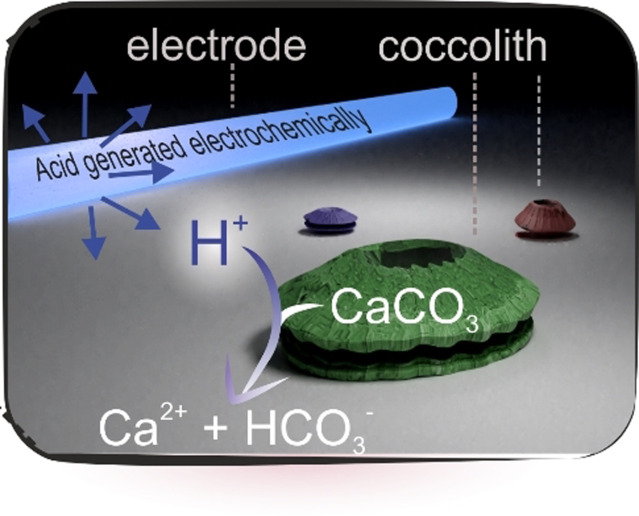

## Introduction

Coccolithophores play a fundamental role in the carbon cycle, producing over 1 billion tonnes (10^15^ g) of calcite and an estimated 6×10^25^ individual coccoliths each year.^[1]^ Coccoliths dominate the calcareous pelagic sediments^[2]^ and are responsible for approximately half of open ocean calcite precipitation.^[1]^ The calcite mass of each individual lith represents the intensity and/or rate of calcite production by coccolithophore cells, and is an important biogeochemical parameter in terms of the impact of coccolithophore production on the alkalinity budget of the surface ocean,^[1]^ which dictates the air‐sea flux of CO_2_.^[3]^ The calcite mass of individual liths also contributes to the export efficiency of organic carbon to depth, the dense calcite mineral aids the ballast of buoyant organic matter *and* prevents the remineralization of the latter by bacteria as it traverses down the water column.^[4]^ In addition to being a fundamental component of the geological cycle of carbon, the mass of secreted coccoliths is also a biologically important characteristic of the cell,^[5]^ and species^[6]^ potentially yielding information about cellular adaptation to growth conditions such as nutrient availability and carbonate chemistry.^[7]^

The secretion of coccoliths onto the surface of a coccolithophore to form an inter‐locking mineralized layer can be at a rate of up to 1–2 per hour.^[8]^ These elaborate bioengineered coccoliths generally, but not exclusively, consist of alternating nano‐units of calcite arranged with the optical axis radial (R units) and vertical (V units) to the plane of the coccolith.^[9]^ In the lifecycle of a coccolithophore, such as the most abundant species *Emiliania huxleyi*, many coccoliths become detached, resulting in a ratio of detached‐coccolith vs. intact‐coccospheres as high as ≈100:1 in surface waters.^[4b]^ Due to the scale of phytoplankton blooms and the light‐scattering properties of calcite, a whitening of surface waters can be observed from space.^[10]^ Satellite studies have revealed that the global coverage of this enhanced scattering in surface‐water, as a result of dense detached liths, is 1.4×10^6^ km^2^ (averaged from 1979–1985).^[11]^ On a global scale, although satellite studies have attempted to convert the remotely‐sensed optical images to maps of calcite mass, this required relating the light‐scattered images remotely acquired from space to local conditions in the surface waters, such as the number and density of coccolithophores and coccolith mass.^[12]^ More generally, to estimate the mass of coccoliths in the open ocean or sediment samples, many use values statistically averaged over large sample sizes, for example, shape factor,^[13]^ which do not account for the natural variability in particulate inorganic carbon per coccolith that inevitably results from changes in coccolithophore diversity and environmental stressors in the marine environment.[^5a^, ^14^] Mass estimation of *individual* coccolith, and the CaCO_3_ content, in the local marine environment will provide a solution to this global challenge which serves as a beacon reporting changes in the marine carbonate chemistry.

**Figure 1:**
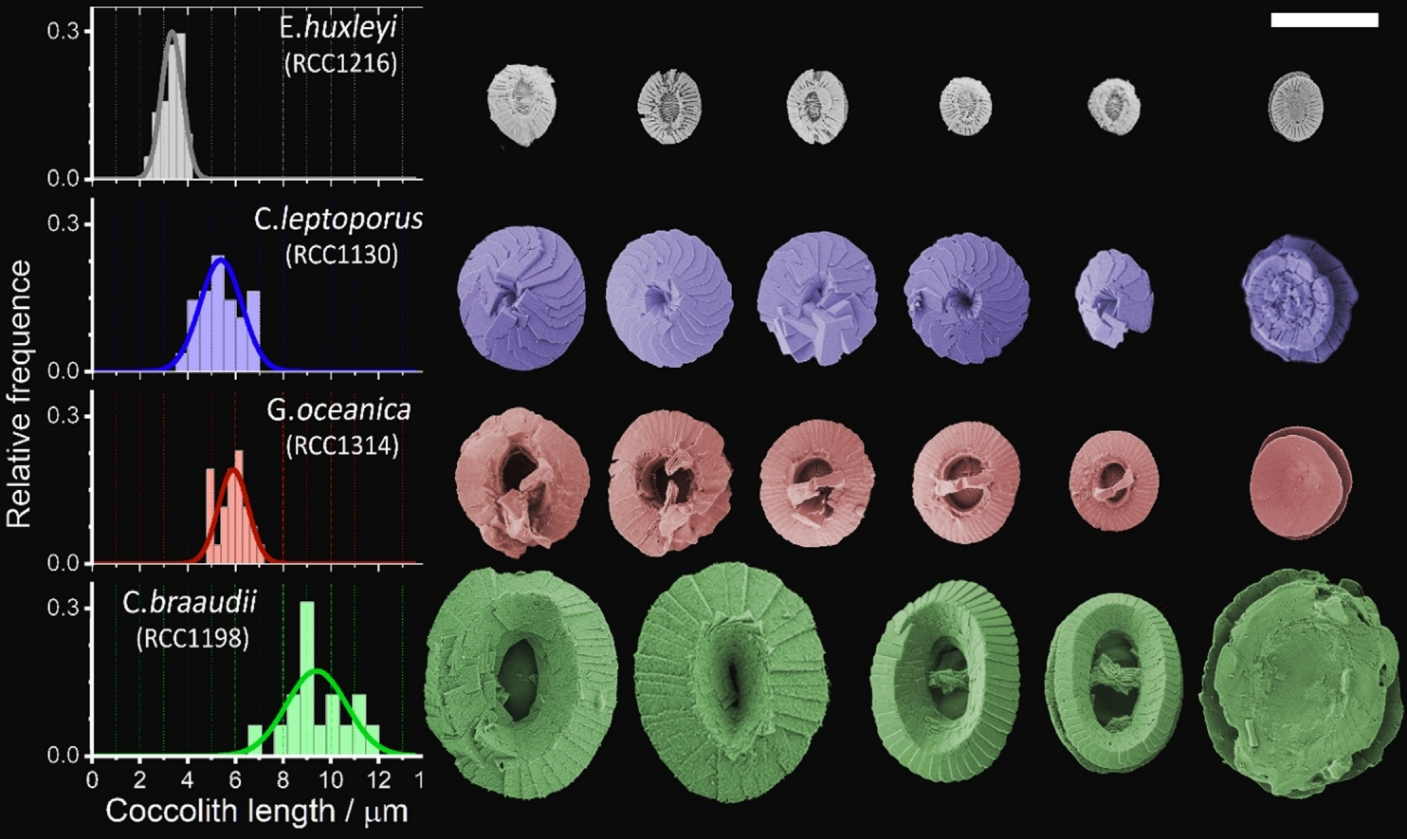
SEM images of detached coccoliths from four species of coccolithophores, namely: *E. huxleyi*, *C. leptoporus*, *G. oceanica* and *C. braaudii*. The histograms on the left show the distribution of coccolith lengths as measured from SEM images. Scale bar=5 μm.

Bio‐accumulated calcium carbonates in the form of coccoliths excreted by phytoplankton vary markedly between species exhibiting significant intra‐species diversity, as shown in the SEM images in Figure 1. This image depicts loose/detached coccoliths from four globally significant species of coccolithophores (*E. huxleyi*, *Calcidiscus leptoporus*, *Gephyrocapsa oceanica* and *Coccolithus pelagicus subsp. braaudii*). These four species reflect the differing sizes, shapes and morphology of the bio‐accumulated calcium carbonate. Due to the small coccolith sizes (Figure 1), the mass of each individual is too small for accurate weight measurement (pico‐ to nano‐grams) using traditional methods. Accordingly, the mass of individual liths is such a biogeochemically and biologically important parameter that a number of methods have been developed for this measurement. One approach is high‐resolution *x*‐ray nanotomography that allows coccoliths to be 3D reconstructed.^[15]^ Alternatively, circular polarised light techniques, which have become popular over the past decade, are restricted to species which have a maximum calcite thickness of 1.56 μm for imaging with a black‐and‐white camera or <4.5 μm in colour.^[16]^ These techniques utilize the birefringence property of calcite crystalline such that retardation of the polarised light emerging from the crystal provides information on crystal thickness.^[16a]^ Prior to measurement, a calibration for pixel‐intensity to calcite thickness is necessary which requires a special cylindrical‐shaped coccolith called rhabdolith.[^16b^, ^17^] Moreover, for birefringence to occur, the optical axis of the calcite crystalline has to be perpendicular to the incident polarised ray. This is problematic for coccoliths composed of entirely V units (or a mixture of R and V units), as the coccolith can appear entirely (or partially) optically isotropic to the incident ray.^[16a]^

An alternative approach to image particles with light‐scattering properties is dark‐field microscopy. When coupled with in situ electrochemical techniques this optical approach can provide real time visualization of particles of nanometre size undergoing dynamic reactions triggered by a suitable electrochemical potential.^[18]^ Our study uses electrochemistry to trigger the calcite dissolution of individual coccolith which is monitored in situ via dark‐field optical measurements allowing 3D reconstruction of the initial coccolith volume, and the CaCO_3_ content, prior to dissolution. This volume measurement is *independent* of knowledge of the crystalline orientation of the calcite in the coccolith, and *applicable to coccoliths of any mass or thickness*. Analytical “titration” of the calcium carbonate content of individual liths is achieved within tens of seconds by the controlled dissolution of a single coccolith with acid electrochemically generated within an opto‐electrochemical cell.

## Results and Discussion

**Figure 2:**
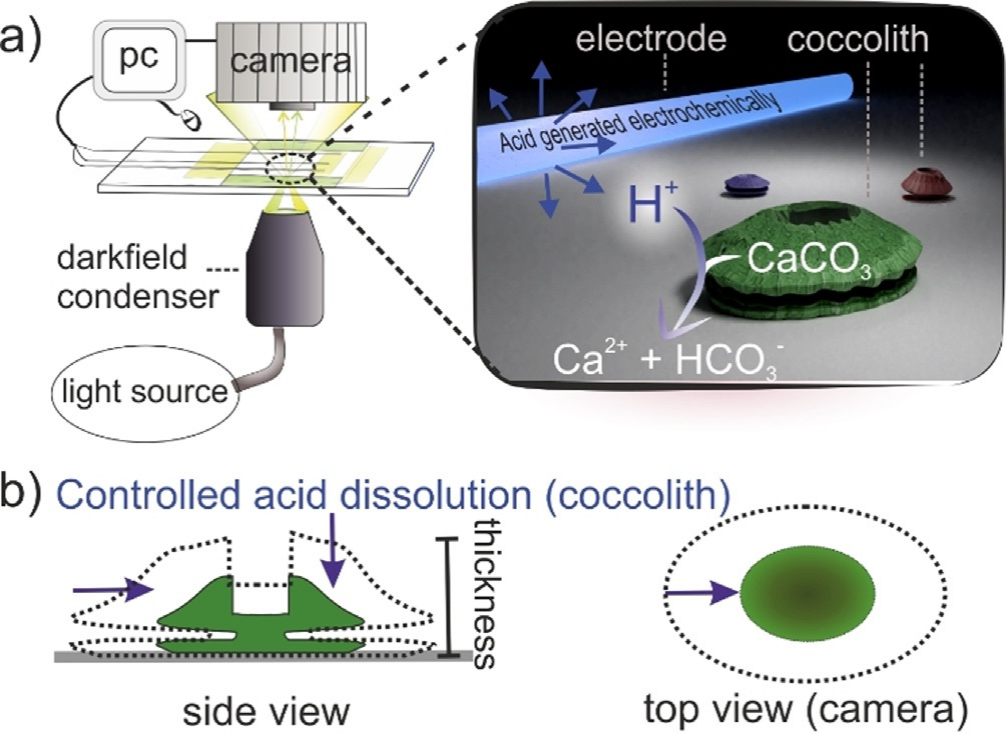
a) Schematic of the opto‐electrochemical experiment setup. At the start of the experiment, acid is generated electrochemically local to the electrode and diffuses radially outward where it encounters the coccoliths. The dissolution of coccoliths under acid attack is monitored via darkfield microscopy. b) dissolution of a coccolith viewed from the coccolith side and top‐down. The purple arrows indicate the coccolith shrinkage as it dissolves under electrochemically induced acid.

In the following, a suspension of detached coccoliths is placed in an opto‐electrochemical cell^[19]^ which allows the generation of a tiny amount of acid local to some of the coccoliths as shown in the schematic in Figure 2. The resulting controlled dissolution of coccoliths is monitored optically, allowing their size to be measured as the dissolution proceeds to complete “titration” of the calcium carbonate content. A series of images recorded during the course of dissolution gives shape and size information, related to the aggregate of bio‐mineralized calcite crystals which constitute the coccolith.

**Figure 3:**
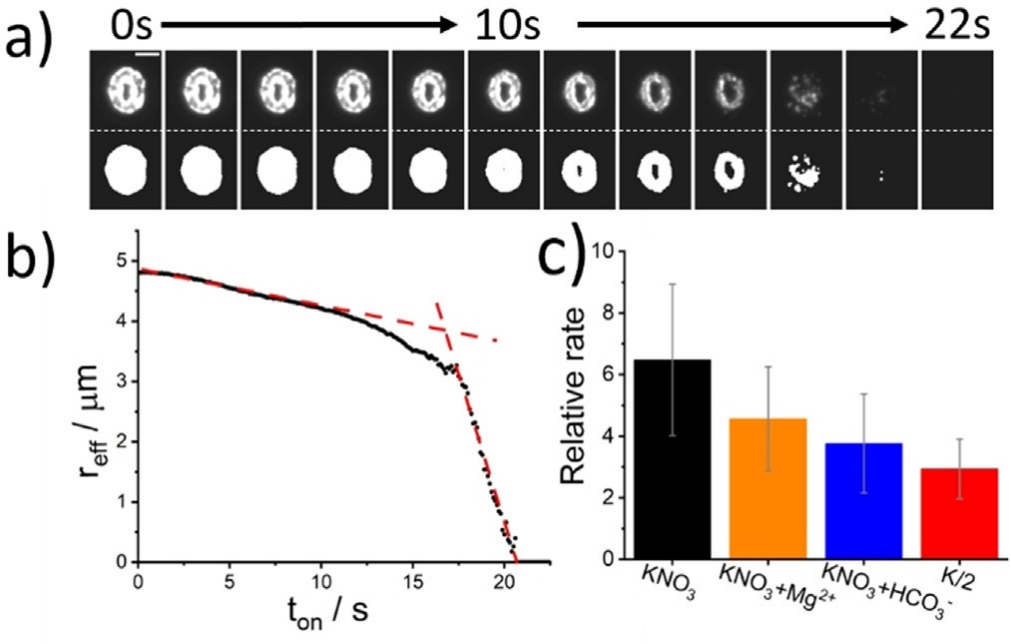
Opto‐electrochemical dissolution of a representative coccolith from C. braaudii. A potential of +1.2 V (vs. Ag wire) was applied to the working electrode at *t*_on_=0 s. a) Temporal evolution of the coccolith optically imaged via dark‐field scatter—top: raw image, bottom: image after auto‐threshold. The coccolith is situated at 45.5 μm from the carbon fibre electrode, measured from the lith centre to the electrode edge. The electrolyte solution was K/2 culture medium with 10 mM H_2_BQ(aq). The time interval of the images is 2 seconds and the scale bar is 5 μm. b) A plot of the effective radius of the coccolith versus time. c) Dissolution rates measured in different electrolyte medium relative to that predicted for smooth and non‐porous calcite particles (see text). Bars: black −0.7 M KNO_3_, orange −0.7 M KNO_3_ + 54.6 mM Mg^2+^, blue −0.7 M KNO_3_ + 2.4 mM HCO_3_^−^ and red—K/2 culture medium.

Specifically, a plankton sample containing coccoliths was mixed with a tiny quantity of electroactive acid precursor (10 mM 1,4‐dihydroxybenzene, H_2_BQ), the sample was placed in a thin‐layer electrochemical cell and the individual coccoliths monitored by dark‐field optical microscopy. The thin‐layer electrochemical cell consists of a three‐electrode setup,^[20]^ including a carbon fibre working electrode (diameter, 7 μm) in a cell volume of approximately 1 cm×1 cm×100 μm (Figure S2). The optical image acquisition of the coccolith was obtained using a 40× objective lens and was digitally synchronized with the electrochemical system. SI sections 1 and 2 provide further details regarding the experimental design and setup. During the experiment, oxidation of the acid precursor (+1.2 V vs. Ag wire) leads to a decrease in the pH local to the working electrode and this drives the dissolution of calcium carbonate.

(1) $$H{}_{2}\mathrm{BQ}-2\;e{}^{-}\to \mathrm{BQ}+2\;H{}^{+}$$

where BQ is benzoquinone. SI section 3 provides more details on the electrochemistry of H_2_BQ. Figure 3 a depicts the temporal evolution of a representative coccolith detached from *C. braaudii* and imaged using darkfield microscopy. The electrolyte is the K/2 growth medium^[21]^ in which the cells were grown. This coccolith was located at a distance of 45.5 μm away from the electrode measured from electrode edge to lith centre and undergoes acid dissolution; the top row of images are obtained directly from a monochrome camera and the bottom images are after image thresholding. The thickness of placoliths are known to be heterogeneous across the lith and are generally the thinnest at the centre.[^9a^, ^15^] This pattern is replicated in the light scattered by the coccolith. At the start of the experiment, a weak but non‐zero pixel intensity varying radially from the centre is seen. By thresholding the optical images, the projection area of the coccolith is obtained irrespective of its thickness nor light intensity. As the experiment proceeds, the coccolith is seen to dissolve over the course of tens of seconds. For coccoliths at a distance to the electrode that is comparable to the coccolith length, that is, tens of microns, the near side of the lith to the electrode is seen to dissolve faster than the far side due to the high concentration gradient of acid near the electrode. In the following, we demonstrate how quantitative kinetic and hence volume information can be obtained from the analysis of these image series.

Placoliths are quasi‐spherical to elliptical in shape, where the shape is specific to the speciation of the coccolithophore^[22]^ as seen in Figure 1. The overall rate of dissolution was inferred by an assessment of the changes in the “effective radius” with time, where π*r*_eff_^2^ is the equivalent projection area of the coccolith. This effective radius was determined from the thresholded images and is plotted against time in Figure 3 b). The changes in the effective radius of the coccolith with time, as inferred from the projection area, provides a dissolution rate averaged over all sides of the coccolith in spite of the acid concentration gradient across the coccolith. Two regimes for the coccolith dissolution in Figure 3 b) are outlined by red lines; a slow initial rate (d*r*_eff_/d*t*=0.056 μm s^−1^) followed by a “rapid” loss of material (d*r*_eff_/d*t*=0.95 μm s^−1^) as the reaction completes (*r*_eff_=0). This change in the optically measured dissolution rate was observed to be a general feature of coccolith dissolution under these conditions and reflects the fact that the coccoliths are disk‐like and are significantly thinner in the dimension perpendicular to the image plane. SI section 4 presents further example dissolution transients all sharing the same qualitative dissolution behaviour. Hence, as the coccolith is dissolved, the *apparent* dissolution rate increases rapidly towards the end of the reaction as the coccolith thickness decreases to a point at which it is no longer identifiable in the optical image. The time taken for the dissolution to occur is therefore more related to the calcite disc thickness, rather than volume. However, prior to discussing the method of deducing the coccolith volume, we first seek to answer the following: what are the dominant chemical factors controlling the coccolith dissolution rate under strong acid attack?

### CaCO3 Dissolution Kinetics in Strong Acid

In multiple opto‐electrochemical experiments, the initial dissolution rate of coccoliths was seen to decrease with an increase in distance of the lith from the electrode, as shown in SI section 4. Close to the electrode (ca. 70 μm) the dissolution starts almost immediately after the start of the proton generation (onset/potential switched on) and the dissolution rate d*r*_eff_/d*t* is linear over the time range of 0–3 s. For larger electrode to lith distances, progressively longer “lag time (s)” are observed. A ca 2.5 s delay before the onset of coccolith dissolution was seen at a coccolith distance of 120 μm from the electrode (Figure S5).

Oxidation of 10 mM of H_2_BQ leads to the formation of a ca. millimolar concentration of protons generated at the electrode interface, resulting in a highly acidic chemical environment (pH<3) local to the wire electrode. The acid, H^+^, diffuses radially outward. The proton concentration profile varies both as a function of time *t* and distance *x* from the electrode [H^+^](*x*,*t*) and is modelled in SI section 5. Importantly, for the electrochemical cell geometry used, in the vicinity (≈50 μm) of the electrochemical interface, a near steady‐state mass‐transport regime is established within ≈1 second, shown in Figure S6. The time required to approach this regime increases progressively with the distance away from the electrode resulting in a delay in the dissolution onset. This explains experimental observations of a “time lag” in the dissolution of coccoliths at a long distance from the electrode (see Figure S5), in which, as noted above, a finite time is required for protons to diffuse hundreds of microns from the electrode. Hence, under the present conditions and to a good approximation, although the proton concentration varies as a function of the distance from the wire, within a distance of 70 μm from the electrode, as evidenced in Figure S6 and Figure S10, the unperturbed acid concentration profile can be considered essentially constant over the experimental time of interest.

When a coccolith is exposed to acid, the calcium carbonate is dissolved in accordance with the following reaction;

(2) $$\mathrm{CaCO}{}_{3}\left(s\right)+H{}^{+}\left(\mathrm{aq}\right)\overset{k{}_{1}}{\underset{}{\to }}\mathrm{Ca}{}^{2+}\left(\mathrm{aq}\right)+\mathrm{HCO}{}_{3}{}^{-}\left(\mathrm{aq}\right)$$

(3) $$\mathrm{HCO}{}_{3}{}^{-}\left(\mathrm{aq}\right)+H{}^{+}\vec{\leftarrow }H{}_{2}\mathrm{CO}{}_{3}\left(\mathrm{aq}\right)$$

(4) $$H{}_{2}\mathrm{CO}{}_{3}\left(\mathrm{aq}\right)\overset{\leftarrow \;\;}{⟶}H{}_{2}O\left(l\right)+\mathrm{CO}{}_{2}\left(g\right)$$

where *k*_1_ is the heterogeneous rate constant as defined by flux=*k*_1_[H^+^]. A value of *k*_1_ equal to 0.043 cm s^−1^ has been reported for the dissolution of a macro‐sized Icelandic Spar (calcite) crystal at pH<4, measured in a buffer‐free electrolyte solution.^[23]^ For an isolated CaCO_3_ particle the rate‐determining step for the dissolution reaction may *either* be the rate of diffusion of protons to the mineral interface *or* the surface reaction rate depending on *k*_1_ and the size of the CaCO_3_ particle. For a *smooth* and solid (non‐porous) calcium carbonate particle, this switch in the kinetic regime occurs at a particle radius of ≈20 μm; as estimated via numerical simulation and is discussed in more detail in SI section 6. For a smooth calcite particle placed in a solution containing a homogeneous acid concentration, [H^+^]_solution_, the mass transport of H^+^ from the bulk solution to calcite particles of radius <20 μm is fast as compared to the rate of consumption at the particle interface, as evidenced in Figure S7 where [H^+^]_surface_≈0.5[H^+^]_solution_. However, as particle sizes shift to much bigger than 20 μm, the mass transport of H^+^ is insufficient to replenish the acid consumed at the calcite interface. Consequently, the rate of acid dissolution is limited by the mass transport of protons.

It is important to recognize that the surface area of a coccolith is higher compared to a smooth, non‐porous calcite particle of the same radius. This non‐unity in the surface roughness will affect the particle size at which the calcite dissolution rate switches from mass‐transport to surface‐area limitation. Figure S8 shows a plot of [H^+^]_surface_ at steady‐state when a calcite particle with non‐unity surface roughness factors is exposed to strong acid solution. The effect of a high surface roughness causes the switch in kinetic regimes to occur at a slightly lower particle radius. In this study, the largest coccoliths are produced by *C. braaudii*, with a typical thickness of 1–2 μm and *r*_eff_≈5 μm. Due to the small dimensions of the coccolith, 1–5 μm when viewed as a calcite disc, the reaction kinetics remains in the surface reaction limited regime even with a surface‐roughness factor of 4–8 so that the mass‐transport of protons to the particle is fast and not rate‐determining, thus in this case [H^+^]_surface_≈[H^+^]_solution_. For the other speciation of coccoliths, which have dimensions smaller than the *C. braaudii* (Figure 1), the acid induced dissolution process is again limited by the surface‐area.

The literature reporting the value of *k*_1_ were conducted under idealized conditions (1.0 M KCl) which do not reflect the ionic composition of seawater.^[23]^ The following seeks to elucidate to what extent other components of seawater alter the dissolution kinetics.

### Effects of the Chemical Components of Seawater on Dissolution Rates

The data presented in Figure 2 b) was measured in the K/2 growth medium. This growth medium contains a variety of salts, trace metals, minerals and a carbonate buffer as tabulated in the SI. In this section, we seek to understand to what extent these constituents might influence the coccolith acid dissolution rate. To this end, the coccoliths were transferred to electrolytes of differing composition. Two primary factors that must be considered when assessing the calcite dissolution kinetics in seawater are i) the influence of Mg^2+^ on the surface reaction rate^[24]^ and ii) the role of the carbonate buffer.^[25]^ Accordingly, individual detached coccoliths (*C. braaudii*) were additionally optical monitored in solutions separately containing i) 0.7 M KNO_3_, ii) 0.7 M KNO_3_ and 54.6 mM Mg^2+^ and iii) 0.7 M KNO_3_ and 2.4 mM HCO_3_^−^. From the thresholded images an effective particle radius was extracted as a function of time. Under all conditions, the plot of particle effective radius versus time again exhibited two distinct linear regimes where the initial rate (μm s^−1^) directly reflects the calcite dissolution kinetics under the prevailing conditions. On the basis of the known distance of the coccolith from the electrode it is possible to compare this initial dissolution rate to that calculated for a smooth, solid and non‐porous calcite particle of the same size dissolved in an ionic solution of 0.7 M KNO_3_. Figure 2 c) plots the measured initial coccolithophore dissolution rates relative to the calculated rate for a smooth and solid particle. Opto‐electrochemical dissolution of detached *C. braaudii* coccoliths in 0.7 M KNO_3_, revealed an initial dissolution rate 6.5(±2.5) times faster than that predicted for a similarly sized “smooth” calcite particle at equal distances from the electrode (see Figure 3 c). This increase of ≈6 in the dissolution rate as compared to that seen for smooth‐surfaced particles reflects the surface roughness of the bio‐excreted coccolith; the dissolution rate is proportional to the calcite surface area and this enhancement of ≈6 is consistent with reports in the literature for the roughness of comparable coccoliths.^[15]^ Performing a similar dissolution experiment with the addition of 54.6 mM Mg^2+^, which correspond to the Mg^2+^ level in seawater, leads to the dissolution rate changing to a relative dissolution rate of 4.5(±1.7). Mg^2+^ cations are known to adsorb on to the calcite surface and inhibit the rate of calcite dissolution and precipitation.^[24]^ Furthermore, the separate addition of bicarbonate (2.4 mM) to the system decreases the dissolution rate to give a relative dissolution rate of 3.8(±1.7). Clearly, the presence of the bicarbonate anion in solution, p*K*_a_ (H_2_CO_3_*)=6.3, serves to partially “titrate” away a fraction of the electrogenerated protons to form its conjugate acid H_2_CO_3_(aq) (see above) which chemically decomposes to form H_2_O(l) and CO_2_(aq). Hence the removal of the protons from the system leads to a concomitant decrease in the dissolution rate of coccoliths. Finally, experiments conducted in the phytoplankton culture medium, K/2, shows a relative dissolution rate of 2.9(±1.0), which is approximately a factor of two lower than that compared for a coccolith in 0.7 KNO_3_. Since the two ions, HCO_3_^−^ and Mg^2+^, serve to decrease the dissolution kinetics via non‐competing mechanisms (solution phase titration and surface inhibition), within error, the decreased dissolution rate in the K/2 medium predominantly reflects the presence of the bicarbonate and magnesium ions with the effects adding to each other.

We conclude, the initial calcite dissolution rate is sensitive to the composition of the ionic solution used, due to the size of the coccoliths, the reaction is limited by the kinetics of the surface reaction. The reaction rate $dr_{eff}$ /dt is essentially constant over the course of the dissolution reaction for a given solution composition. The following section demonstrates how this reaction rate can be used to infer the thickness of the coccolithophore and hence provide a measurement of the volume of individual coccoliths.

### Extracting Coccolith Volume from Dissolution Kinetics

In the opto‐electrochemical cell, the coccolith is exposed to an electrochemically generated acid environment $\left[H^{+}\right]\left(x,t\right)$ and the dissolution reaction occurs almost uniformly across the surface of the coccolith. In the surface‐area controlled kinetic regime, the rate of mass transport is fast and at an electrode distance larger than the coccolith length, the proton concentration at the side/rim of the coccolith is no different to that on the top of the coccolith. Therefore, the initial average dissolution rate, v, is obtained from the orthographic projection of the coccolith on the 2D image plane from d*r*_eff_/d*t*. We assume this rate controls the dissolution of the shortest dimension of the lith which controls the time for complete dissolution by acid titration. The time to dissolve completely is expected to vary between liths (both intra‐ and inter‐species) and to further be sensitive to the prevailing chemical environment. However, for the purpose of lith volume measurement a key fact is that the dissolution rate is limited by the rate of the surface reaction (as opposed to mass‐transport). Consequently, this rate is essentially constant during the course of the dissolution, due to the quasi‐steady‐state proton concentration arising from the electrochemical cell geometry—see SI Section 5 and 6.

**Figure 4:**
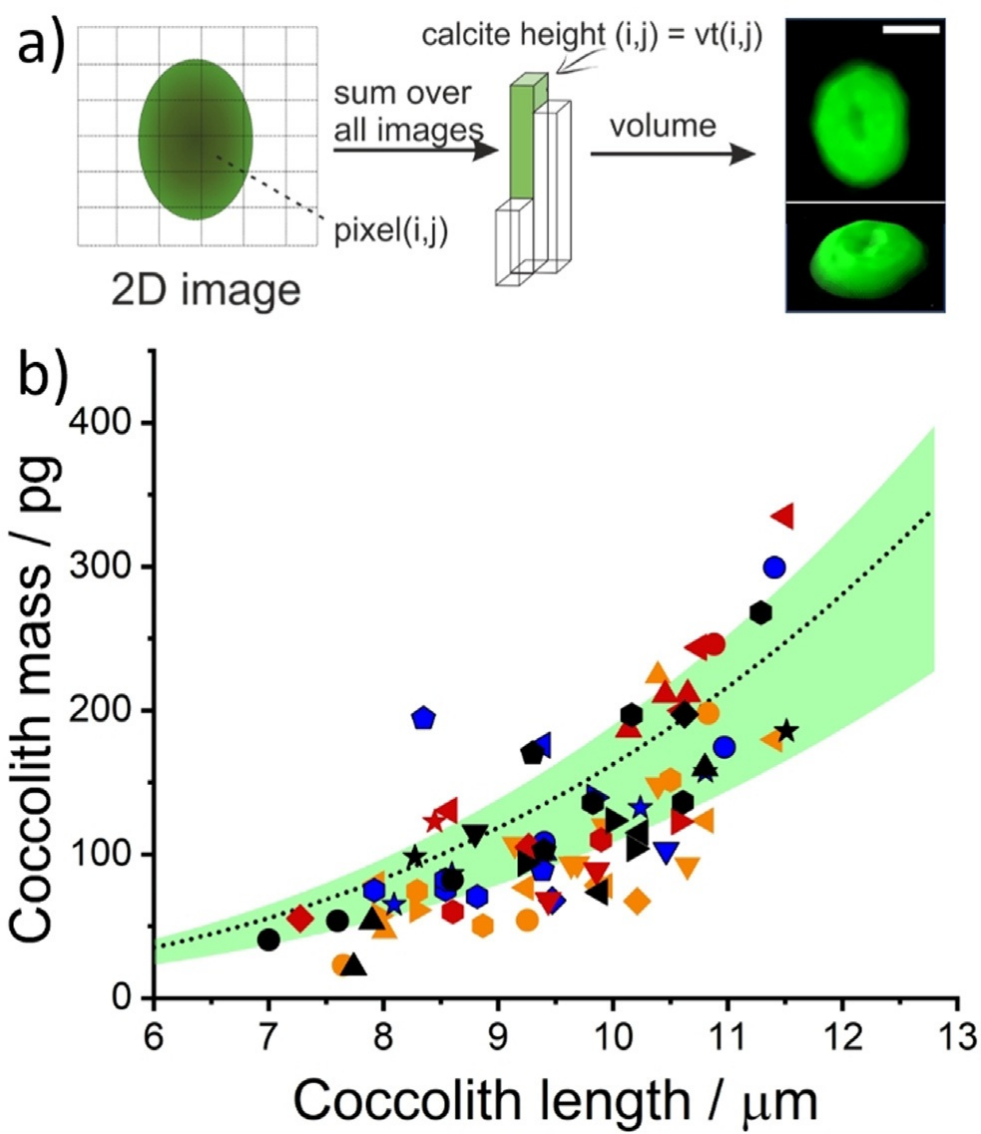
a) Data analysis procedures leading to the reconstructed representation of a *C. braaudii* coccolith dissolved in the opto‐electrochemical experiment. Further detail is provided in SI section 2.4. The temporal evolution of this coccolith during acid dissolution is shown in Figure 2 a) with an initial dissolution rate (*v*=d*r*_eff_/d*t*) of 0.056 μm s^−1^ as shown in Figure 2 b). The electrolyte is K/2 growth medium with 10 mM H_2_BQ acid precursor. Scale bar=5 μm. b) A scatter plot showing the estimated mass of *C. braaudii* coccoliths in various electrolytes, all with 10 mM of H_2_BQ acid precursor added prior to the experiment. The coccoliths within a distance 10–100 μm from the electrode were analyzed. The colour Scheme representing the electrolyte solution is unchanged from Figure 3 c. The different symbols with the same colour represent data from repeated experiments. The overlaid black dotted line is the estimation of coccolith mass using the recommended shape factor (*k*_s_) of 0.06 and green shade is the range of *k*_s_ values (0.04–0.07) reported by Young et al.^[13a]^

Knowledge of the initial rate enables the thickness of the calcite to be determined on a per‐pixel basis. The volume of individual coccoliths can be determined by iterating through the stack of images, building on a pixel‐by‐pixel basis, to yield the initial volume of coccolith prior to dissolution. Figure 4 a) depicts the image analysis procedure leading to a 3D model of an individual coccolith, where the volume was reconstructed from a series of images taken during the course of the dissolution process, shown in Figure 3 a). The image analysis procedure is as follows, for more see SI section 2.4. In step one, as above, we image an individual coccolith during controlled acid dissolution. In step two, as described earlier, the images are analysed to give the projection area of the coccolith as a function of time. Next, step three, the initial dissolution rate (*v*=d*r*_eff_/d*t*) is measured. Step four, the time required to completely titrate the calcite, *t*(*i*, *j*), is measured for each and every pixel coordinates (*i*, *j*). Step five, the calcite height, which is normal to the 2D image plane, is estimated at each pixel coordinate (*i*,*j*) by multiplying *t*(*i*,*j*) by v. Last, by doing this on a per pixel basis the initial thickness of the coccolith can be inferred as a function of pixel position hence yielding a 3D reconstruction of the original coccolith shown in Figure 4 a).

The method of volume extraction used is *independent* of the conditions under which it has been measured, as the initial dissolution rate is used to directly infer the particle thickness from the time to full dissolution. Table S2 contains optical images of four *C.braaudii* coccoliths obtained prior to acid dissolution in different electrolytes and their 3D reconstructed images after controlled acid dissolution. Irrespective of the electrolyte medium used, the reconstructed images of the coccolith resemble that prior to dissolution. Note that the resolution of the 3D coccolith reconstructed via this method is not as high as obtained via atomic force microscopy,^[26]^ electron microscope^[13a]^ or 3D X‐ray nanotomorgraphy^[15]^ due to the nature of optical limitation and the nature of reconstruction from a stack of projection images; the general shape of the reconstructed coccoliths is however fully consistent with the shape of placoliths reported elsewhere in the literature. From the reconstructed individual coccolith volumes, it is possible, assuming a given lith calcite density, to convert the volume to a coccolith mass. Figure 4 b) plots the mass of individual *C. braaudii* coccoliths as measured in the four different electrolyte solutions (separated by colour); different symbols of the same colour represent data obtained from repeated experiments. Notice that the reconstruction is independent of the chemical composition of the electrolyte as diverse solutions are used to generate Figure 4 b). The initial dissolution rate of a coccolith is dependent on the difference in electrolyte chemical composition, the distance of coccoliths from the electrode, and less importantly, the intra‐species variation in coccolith surface roughness and morphology. Since the volume reconstruction process uses the initial dissolution rate as measured, this internally “calibrates” for all of the effects discussed above so that the result is independent of the numerous variables. However, of course, the faster the initial dissolution rate, the quicker the coccolith dissolves and the fewer images there are for the volume reconstruction. The mean mass of *C. braaudii* coccoliths across all four of the electrolyte studied is 0.122(±0.064) ng, sample size=81, which is in excellent agreement with literature values.^[13a]^ The latter was overlaid as green shade and the dotted black line in Figure 4 b).

**Figure 5:**
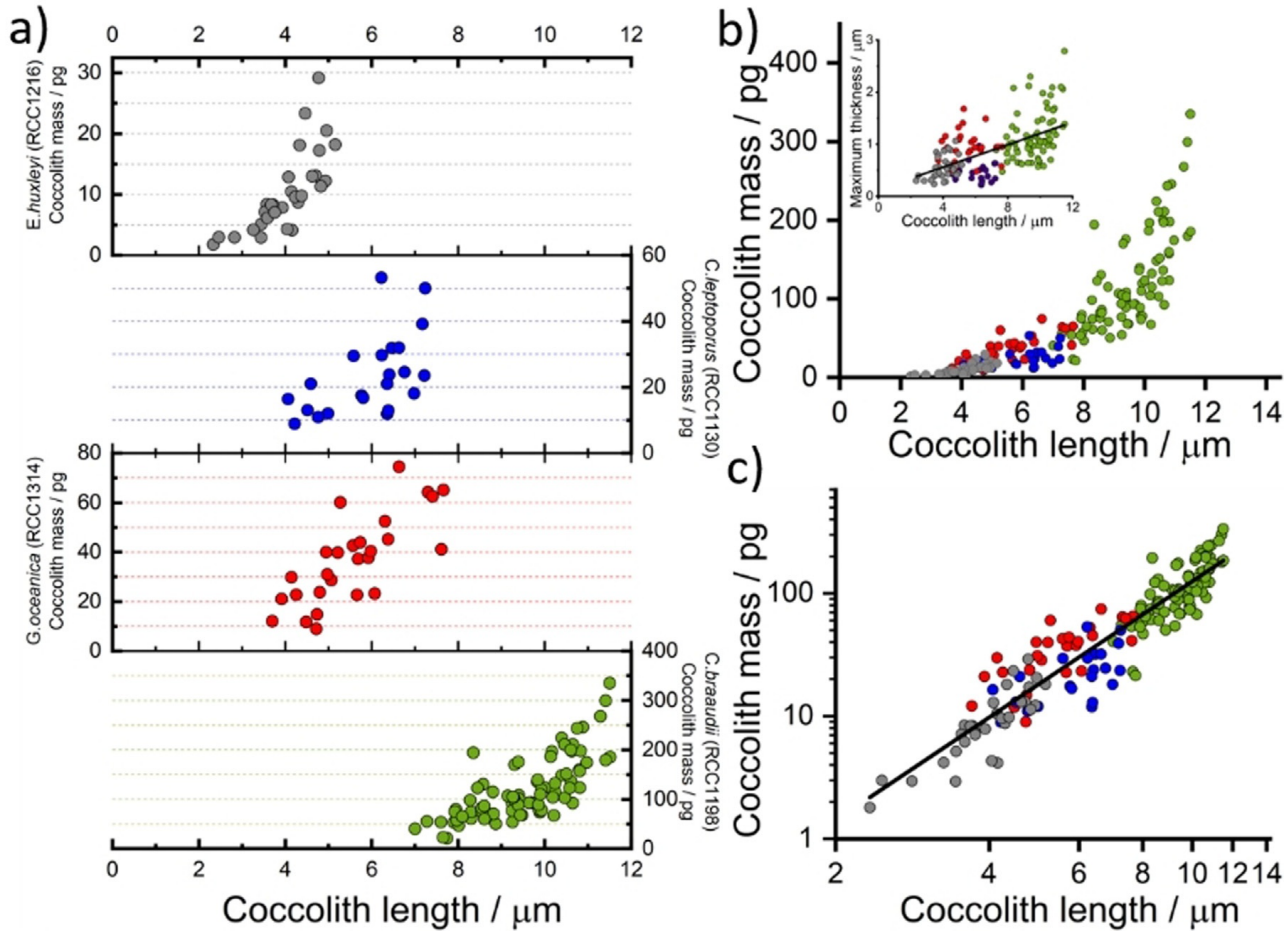
a) Coccolith mass estimated for individual coccoliths from four different coccolithophore species. b) Collective estimated coccolith mass against coccolith length. Inlay: the maximum thickness of coccolith from image reconstruction, Pearson's r=0.58. c) Collective mass plotted on a logarithmic scale. Circles: grey—*E. huxleyi* (RCC1216), blue—*C. leptoporus* (RCC1130), red—*G. oceanica* (RCC1314) and *C. braaudii* (RCC1198). Line of best fit over all data: slope=2.78±0.09 and Pearson's r=0.94. The data for *E. huxleyi*, *G. oceanica* and *C. leptoporus* were measured directed from their respective culturing medium with added 10 mM H_2_BQ acid precursor, whereas those for *C. braaudii* is the collective data from all four electrolytes as seen in Figure 2 d).

Having evidenced the technique using coccoliths detached from *C. braaudii* we next extend this study to other coccoliths. Figure 5 shows the inferred coccolith mass from three additional species of coccolithophore—*E. huxleyi*, *C. leptoporus* and *G. oceanica*. The opto‐electrochemical dissolutions were conducted in their corresponding “seawater‐mimicking” culturing media, with the addition of 10 mM H_2_BQ acid precursor prior to the experiment. The coccolith mass is seen to generally increase with coccolith length inter‐species. The average coccolith mass for *E. huxleyi*, *C. leptoporus* and *G. oceanica* are 10.2(±6.5), 23.6(±12.1) and 37.0(±17.8) picograms (pg) per lith, respectively. *E. huxleyi* (RCC1212) and *G. oceanica* (RCC1314) were recently studied via birefringence polarised light approach and 3D X‐ray coherent diffraction imaging; the reported mass of *E. huxleyi* coccoliths were ca. 1–6 pg for coccolith lengths between 2 to 4 μm, and the *G. oceanica* coccoliths are ca. 5–30 pg for coccolith lengths between 4 to 6 μm.^[15]^ Compared to the coccolith mass obtained via image reconstruction, good agreement are seen within the overlapping range of coccolith lengths. We note that the variation in the coccolith length range in the sample, and the proportion of malformed/broken coccolith, may be due to variations in different culturing conditions and/or experiments conducted at different stages during the coccolithophore lifecycle. The size range of the *C. leptoporus* coccoliths herein (4–7 μm) is small as compared to those generally reported by Young et al. from sediment samples (5–11 μm, average mass=74.1 pg). Moreover, as shown in Figure S1, SEM images revealed a large proportion of the *C. leptoporus* coccoliths in this study are either malformed or broken, which may concatenate with the small size distribution leading to an underweight average coccolith mass of 23.6 pg. Figure 5 b) shows the collective coccolith mass data showing the relationship of coccolith mass versus coccolith length that exists in both intra‐species *and* inter‐species, leading to a linear logarithmic plot as shown in Figure 5 c) with a slope equal to 2.8(±0.1). From this, one can infer the coccolith mass, both intra‐ and inter‐species, varies broadly with the coccolith length cubed. However, as is consistent with the literature and can be seen from the inset of Figure 5 b), the correlation between the measured thickness and the coccolith length is low (Pearson's *r* value of 0.58).^[27]^

## Conclusion

In situ electrochemical dissolution of coccoliths with simultaneous in situ optical image analysis allows the volume and mass of individual coccoliths to be estimated. The dissolution of coccoliths in a strongly acidic environment occurs under a kinetic regime controlled by the particle surface‐area; within this regime, the rate of change in the projection area of the coccolith during dissolution is directly proportional to the rate of change in the coccolith thickness. This allows the coccolith to be reconstructed from the time‐stacked 2D images, to provide an estimate of coccolith volume on a pixel‐by‐pixel basis. Summing across all of the pixels allows the initial pre‐dissolution total coccolith volume to be determined and hence this yields a measure of the CaCO_3_ mass.

The opto‐electrochemical approach uses the initial rate of coccolith dissolution, inferred from d(*r*_eff_)/d*t*. It therefore internally calibrates *all* factors that may affect the dissolution rate; these include: the surface roughness of the coccolith, the distance of the coccolith from the electrode, the presence of inhibitors for calcite dissolution. Since the analysis is performed on an individual coccolith basis, it does not rely on using a quantity statistically averaged over, for example, large sediment samples (e.g. shape factor, *k*_s_). Therefore, the coccolith mass estimated herein could in principle account for sample abnormalities such as those that include a large proportion of deformed, partially dissolved, or broken coccoliths. The opto‐electrochemical method can discriminate coccoliths from suspended sediments, which cannot be done in an operational manner using conventional remote sensing approaches and thus holds the potential to provide new insight into the presence of coccolithophores in the carbon pool of coastal waters, using in situ samples.

Unlike the birefringence methods which have a detection limit in the greyscale limited to a theoretical maximum of 1.56 μm and *require* a cylindrical rhabdolith to calibrate intensity with thickness; the approach herein provides a facile alternative due to the calibration‐free approach and has no in‐principle limitation to the thickness of calcite particle *nor* the crystalline orientation of calcites in the coccolith. This allows such opto‐electrochemical method to probe volumes of larger coccoliths, as evidenced by the *C. braaudii* coccoliths as presented herein, and with the possibility to extend the study to living coccolith‐bearing coccospheres and/or study of sediment samples without prior knowledge of the coccolith species nor thickness of coccoliths within the sample. The scientific breakthroughs presented herein may find use in marine environments reporting changes in the carbonate biogeochemistry as inferred from local changes in coccolith mass, providing a solution to the global challenge.

## Conflict of interest

The authors declare no conflict of interest.

## Supporting information

As a service to our authors and readers, this journal provides supporting information supplied by the authors. Such materials are peer reviewed and may be re‐organized for online delivery, but are not copy‐edited or typeset. Technical support issues arising from supporting information (other than missing files) should be addressed to the authors.

Supporting Information
