# Supplementary material for: Opto‐Electrochemical Dissolution Reveals Coccolith Calcium Carbonate Content
Source: Angew Chem Int Ed Engl. 2021 Aug 15;60(38):20999–1006. doi: 10.1002/anie.202108435 (PMC8518593; doi:10.1002/anie.202108435)
Supplement: Supplementary file 1 — Supporting Information [file ANIE-60-20999-s001.pdf]

Angewandte Chemie
Eine Zeitschrift der Gesellschaft Deutscher Chemiker

Supporting Information

Opto-Electrochemical Dissolution Reveals Coccolith Calcium Carbonate Content

*Minjun Yang, Christopher Batchelor-McAuley, Samuel Barton, Rosalind E. M. Rickaby, Heather A. Bouman, and Richard G. Compton**

anie_202108435_sm_miscellaneous_information.pdf

Table of Contents

Section 1 Chemicals 3

1.1 Electrochemistry 3

1.2 Cell culturing media 3

Section 2 Imaging 5

2.1 Scanning electron microscopy 5

2.2 Optical microscopy 5

2.3 Combined opto-electrochemistry 5

2.4 Image analysis 6

Section 3 Redox chemistry of the acid precursor 1,4-dihydroxybenzene 9

Section 4 Experimental calcite dissolution rate versus distance from the electrode 11

Section 5 Mass-transport of electrochemically generated reagents: numerical simulation 12

Section 6 Calcite particle dissolution kinetics 15

6.1 Surface reaction kinetics 15

6.2 Mass-transport and the effect of particle size 16

6.3 Experimental verification of the dissolution kinetics 19

6.4 Numerically predicted dissolution kinetics of a calcite particle as a function of distance from the electrode 20

Section 7 Coccolith data 22

Section 1 Chemicals

1.1 Electrochemistry

Potassium nitrate was purchased from Scientific Laboratory Supplies Limited, U.K. 1,4-dihydroxybenzene was purchased from Sigma-Aldridge, U.K. Detrital calcium carbonate powder was purchased from Alfa Aesar, U.S. The shape of the received calcium carbonate crystallinity in the $CaCO_3$ powder is rhombohedral under light microscope, which is a strong indication of calcite crystalline. The powder was ground in a pestle and mortar for 10 minutes to obtain the quasi-spherical-shaped calcite shown in Section 2.2. All chemicals were used without further purification. Aqueous solutions were made using ultrapure water (Millipore, resistivity 18.2 MΩ cm at 25 °C). All of the H_2BQ solutions were freshly prepared on the day of the experiments.

1.2 Cell culturing media

The cultures used in this study were obtained from Roscoff Culture Collection (RCC, France): E.*huxleyi* (RCC1216), G.*oceanica* (RCC1314), C.*leptoporus* (RCC1130) and C.*braarudii* (RCC1198). All cultures were maintained by regular batch culturing on a K/2 enriched growth medium modified from the recipe for K medium by Keller *et al.* with f/2 vitamins and silica component omitted.[1] Aquil synthetic ocean water was used instead of natural seawater.[2] The final molarity of each of the medium components in the K/2 recipe are summarized in Tables S1. To maintain the cultures, regular batch culture inoculations were made into Tissue Culture Treated Flasks (culture area 25 cm^2, Corning™ Falcon™, USA), under sterile conditions, to a total culture volume of 40ml. All cultures were kept in a PHCbi MLR-352-PE Incubator (PHC Eurpe B.V.), on a 14:10 light – dark cycle with a PAR intensity of 40-60 µmol m^{-2} s^{-1}. For the electrochemical dissolution experiments, coccolith samples in their respective late stationary phase were studied. This resulted in proportionally more detached coccoliths to complete coccospheres in the culture medium.

Table S1 : A summary of the molar concentration of all the components in the synthetic ocean water based K/2 growth medium.

		Molar Concentration in final K/2 growth medium (mol dm^{-3})
Synthetic Ocean Water (SOW) based on the Aquil medium recipe[2]	NaCl	4.20×10^{-1}
	Na_2SO_4	2.88×10^{-2}
	KCl	9.39×10^{-3}
	$NaHCO_3$	2.38×10^{-3}
	KBr	8.40×10^{-4}
	H_3BO_3	4.85×10^{-5}
	NaF	7.15×10^{-5}
	$MgCl_2 . 6H_2O$	5.46×10^{-2}
	$CaCl_2.2H_2O$	1.05×10^{-2}
	$SrCl_2.6H_2O$	6.38×10^{-5}
K/2 medium enrichment based on the K recipe,[1]	$NaNO_3$	4.41×10^{-4}
	NH_4Cl	2.50×10^{-5}

with silica component omitted	Na_2 b-glycerophosphate	5.00 x 10^{-6}
	H_2SeO_3	5.00 x 10^{-9}
	$Na_2EDTA.2H_2O$	5.55 x 10^{-5}
	$FeCl_3.6H_2O$	5.85 x 10^{-6}
	$MnCl_2.4H_2O$	4.50 x 10^{-7}
	$ZnSO_4.7H_2O$	4.00 x 10^{-8}
	$CoCl_2.6H_20$	2.10 x 10^{-8}
	$Na_2MoO_4.2H_2O$	1.30 x 10^{-8}
	$CuSO_4.5H_2O$	5.00 x 10^{-9}
F/2 vitamin enrichment[2]	Thiamine. HCl (Vitamin B1)	2.96 x 10^{-7}
	Biotin (Vitamin H)	2.05 x 10^{-9}
	Cyanocobalamin (Vitamin B12)	3.69 x 10^{-10}

Section 2 Imaging

2.1 Scanning electron microscopy

The phytoplankton culture containing the coccoliths were filtered using a poly-carbonate filter (0.01 µm), washed with deionized water, and subsequently dried at atmospheric pressure. Prior to the imaging, 10 nm of gold thin-layer was coated over the sample using a rotary pumped coater (Q150RES, Quorum, UK). The Scanning Electron Microscopy (SEM) images were obtained using a Sigma 300 FEG-SEM from Zeiss with an accelerating voltage of 2.0 kV.

Figure S1 presents a representative image of the C.*leptoporus* coccoliths, as highlighted in the main body a number of the coccoliths were found to be deformed.

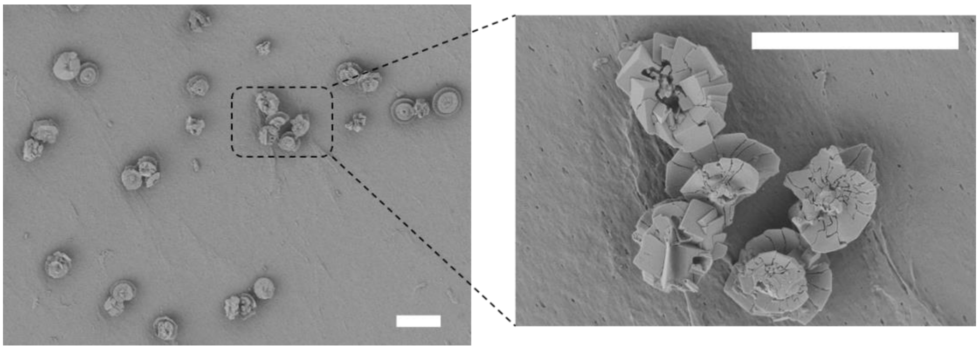

*Figure S1: SEM images of coccoliths detached from C.*leptoporus. Note the presence of some deformed coccoliths. Scale bar = 10 µm.

2.2 Optical microscopy

Optical measurements were made on a Zeiss Axio Examiner, A1 Epifluorescence microscope (Carl Zeiss Ltd., Cambridge U.K.), using a 40x oil immersion objective (Plan-Apochromat 40x/1.3 Iris (UV)VIS-IR). The objective lens aperture was set to 0.7 to optimize for darkfield contrast. The darkfield illumination of coccoliths was achieved by using an oil-immersion darkfield condenser (NA = 1.2/1.4, Carl Zeiss Ltd., Cambridge U.K.). The video acquisition was provided by a Hamamatsu ORCA-Flash 4.0 digital CMOS camera (Hamamatsu, Japan), providing 16-bit images with 4-megapixel resolution.

2.3 Combined opto-electrochemistry

The opto-electrochemical cell is built on top of a glass slide to allow the acid dissolution of individual coccoliths to be imaged during the experiment. Figure S2 shows a schematic of the thin-layer cell used. The cell consists of two carbon fibre electrodes (diameter 7µm, Goodfellow Cambridge Ltd.) which act as working and counter electrodes. A third electrode, a silver wire (diameter 50µm, Goodfellow Cambridge Ltd.) is used as the pseudo-reference electrode. The cell depth is roughly 100 µm. Potentiostatic control and synchronization with the camera were provided by an previously developed in-house built device[3] and current-amplifier (Keithley 427) from Keithley Instruments Inc, US. All opto-electrochemical experiments were all conducted at room temperature.

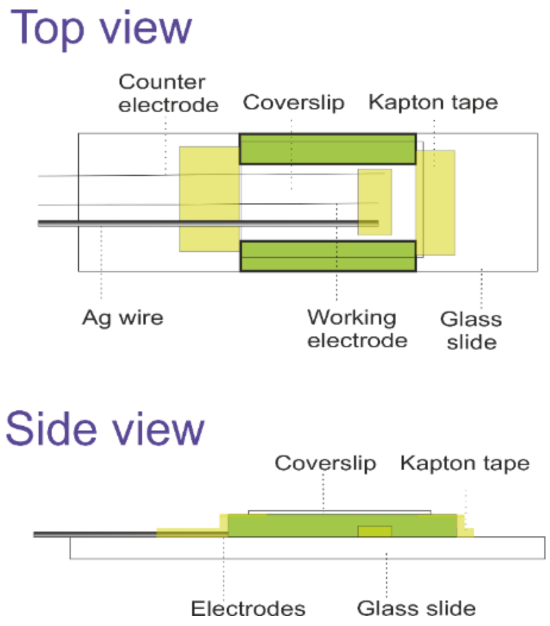

Figure S2: Schematic of the bespoke opto-electrochemical cell. The cell has three electrodes: a working, counter and reference electrode. The working and counter electrode are carbon fibre wires (r_e=3.5µm), whereas the reference is an Ag wire electrode (r_e = 25µm). The cell depth is approximately 100µm.

2.4 Image analysis

During the course of the opto-electrochemical experiment the cell was optically imaged at 10 fps. The resulting series of images were analyzed using (Fiji) ImageJ freeware.[4] Macro scripting in ImageJ allows the coccoliths to be identified and the distance from the working electrode to be measured, reported as electrode edge to coccolith centre. The stack of images containing individual coccoliths were cropped and duplicated to allow auto-threshold ('Huang2') over the stack histogram to be performed for each of the individual coccoliths. 'Huang2' is a built-in implementation allowing systematic image thresholding without input from the user. The projection area of a coccolith is found by counting the total number of white pixels in the binary 2-D image multiplied by the pixel resolution (0.155x0.155 μm^2 $pixel^{-1}$). To estimate the volume of a coccolith, the number of white pixels in the z-stack of binary images as obtained during the coccolith dissolution is summed across each of the image coordinates in the x-y directions – illustrated in Figure S3. The number of white pixels in the z-stack, at each x-y image coordinate, is then multiplied by the initial dissolution rate (obtained experimentally, $\mu m\ s^{-1}$) and the time between each image to reveal the reconstructed coccolith as shown in Figure S3 (shaded green). Assuming the coccolith is entirely made of calcite, the mass of each coccolith is estimated by multiplying the estimated volume by the density of calcite (2.71 g cm^{-3} at 25°C)[5].

Notice that the image reconstructed C.*braaudii* coccolith in Figure S3 resembles the shape of that obtained in SEM images. The finer structural detail of the coccolith is not comparable to imaging using electron microscopy due to the limitation of optical resolution and the nature of reconstruction from a stack of projection images. But in terms of the estimation of coccolith mass, as shown in the main manuscript, this method reconstructs

coccoliths with mass values spanning over 2 orders of magnitude, successfully showing both intraspecies and interspecies variation. Table S2 shows four optical and reconstructed images of *C.braaudii* coccolith before and after dissolution in different electrolyte media.

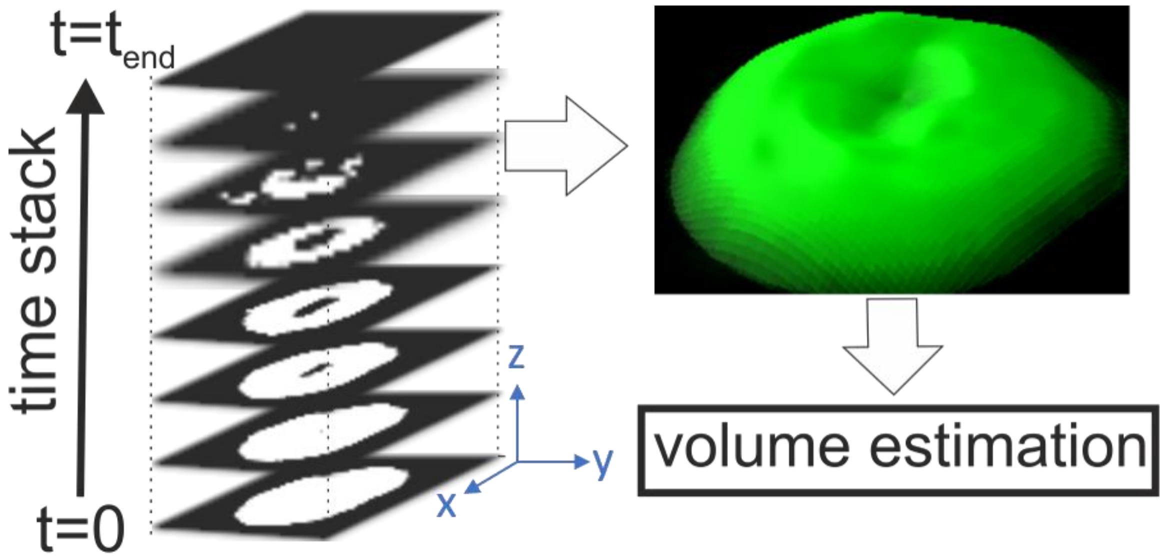

Figure S3: A schematic showing the image reconstruction process in estimating the volume of the coccolith prior to dissolution. All image analysis were performed in Fiji ImageJ freeware.

SUPPORTING INFORMATION

Table S2. Optical and reconstructed images of *C.braaudii* coccoliths from different electrolytes, obtained prior to and after acid dissolution, respectively.

Electrolyte	Distance from electrode (μm)	Raw image (t=0)	Reconstructed image after acid dissolution	Reconstructed image in 3D view
**KNO_3**	27.1	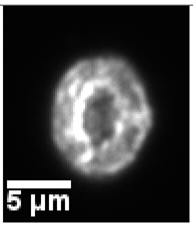	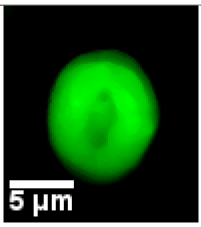	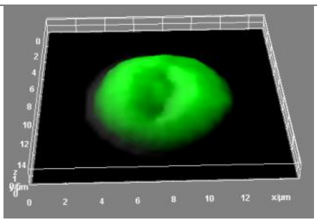
**$KNO_3 + Mg^{2+}$**	23.2	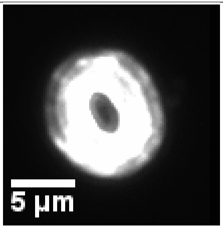	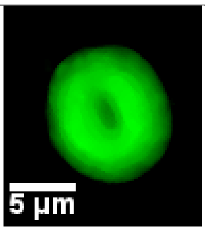	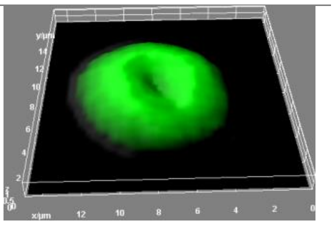
**$KNO_3 + HCO_3^-$**	34.7	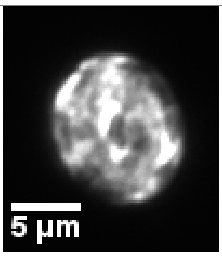	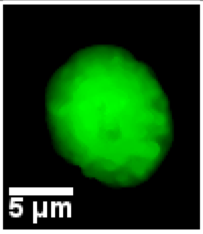	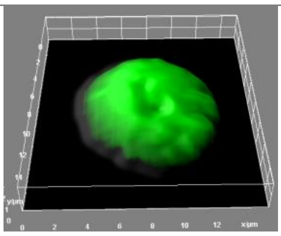
**K/2**	26.8	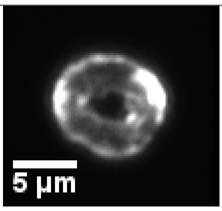	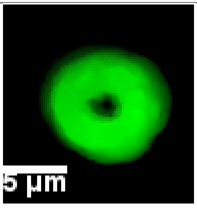	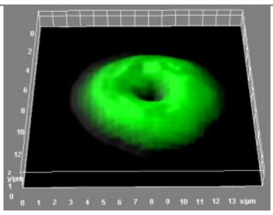

Section 3 Redox chemistry of the acid precursor 1,4-dihydroxybenzene

1,4-dihydroxybenzene (H_2BQ) is a redox-active species able to undergo a two-electron oxidation leading to the formation of benzoquinone (BQ) in aqueous solution. The first and second pK_a's of H_2BQ at 25°C are 9.85 and 11.84, respectively.[6] Consequently at around neutral pH the H_2BQ is fully protonated. Consequently, as shown in the expression below oxidation of the H_2BQ leads to the release of two protons.

$$H_2BQ(aq) - 2e^- \rightleftharpoons BQ(aq) + 2H^+(aq) \quad \text{Eq. S1}$$

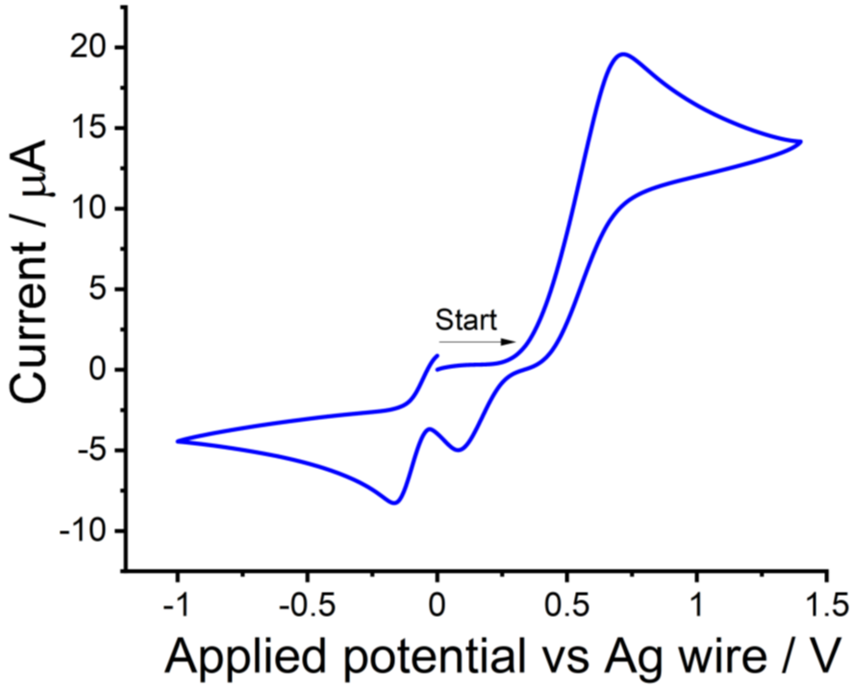

Figure S4: Cyclic voltammogram of 10mM H_2BQ in 0.7M KNO_3 recorded in the opto-electrochemical cell at a voltage scan rate of 50mVs^{-1}. The working electrode is a carbon fibre wire electrode (radius = 3.5 µm, length ≈ 1cm). The potential was swept anodically from a starting potential of 0.0V to +1.4 V vs Ag wire before reversing the sweep direction to -1.0V and stopped at 0.0V.

Figure S4 shows a cyclic voltammogram of 10mM H_2BQ recorded in 0.7M KNO_3 electrolyte, recorded in the above discussed opto-electrochemical cell. The potential was swept from an initial potential of 0V anodically to +1.4V (vs Ag wire) at a voltage scan rate of 50mVs^{-1}. A clear oxidative peak corresponding to the oxidation of the H_2BQ occurs at ~+0.7V (vs Ag wire). In the absence of pH buffering species, although the electrolyte is initially neutral in pH the electrode interface quickly becomes highly acidic due to a buildup of protons generated electrochemically (Eq. S1). Thus, the forward anodic H_2BQ/BQ peak seen at +0.7V (vs Ag wire) in Figure S4 is similar to that reported in pH 2.0 buffered electrolyte.[7] From the cyclic voltammogram, it is clear that at an applied over-potential of +1.2V (vs Ag wire) the 2-electrons oxidation of H_2BQ is fully driven to generate BQ and 2 stoichiometric equivalents of protons.

In this work the 1,4-dihydroxybenzene is used as an acid precursor which upon oxidation leads to the release of protons and hence a decrease in the pH local to the electrode. The spatial and temporal evolution of [H^+] in the opto-electrochemical cell is discussed in SI Section 5.

Section 4 Experimental calcite dissolution rate versus distance from the electrode

As discussed in Section 3, the pH gradient in the opto-electrochemical experiments is a function of both time and distance of coccolith from the electrode. Figure S5 shows the temporal change in the effective radius of C.*braaudii* coccoliths situated at different distances from the electrode. The experiment was conducted in a buffer-free 0.7M KNO_3 electrolyte solution. The y-axis shows a relative change in the effective coccolith radius as compared to that measured prior to the acid dissolution ($t_{pot.on}$ = 0s) so as to normalize for intra-speciation size differences. For coccoliths within ~70µm from the electrode, two linear slopes can be seen for the reasons discussed in the main text. An initial slope (µm s^{-1}) can be extracted from the initial 3 seconds of dissolution, as can be seen in Figure S5 b); generally, the further the coccolith is away from the electrode, the initial dissolution rate is seen to be slower and requires a longer time for complete dissolution (r_{eff} = 0). For coccoliths much further away from the electrode, such as the one at 120 µm shown in Figure S5 however, a delay of ~2.5s can be seen before the dissolution is initialized by electrochemically generated acid. This is entirely consistent with the diffusion of protons from the electrode interface outwards into the solution, in which a finite time is required for an appreciable amount of protons to reach a distance of hundreds of microns from the electrode – shown in SI Section 6.4.

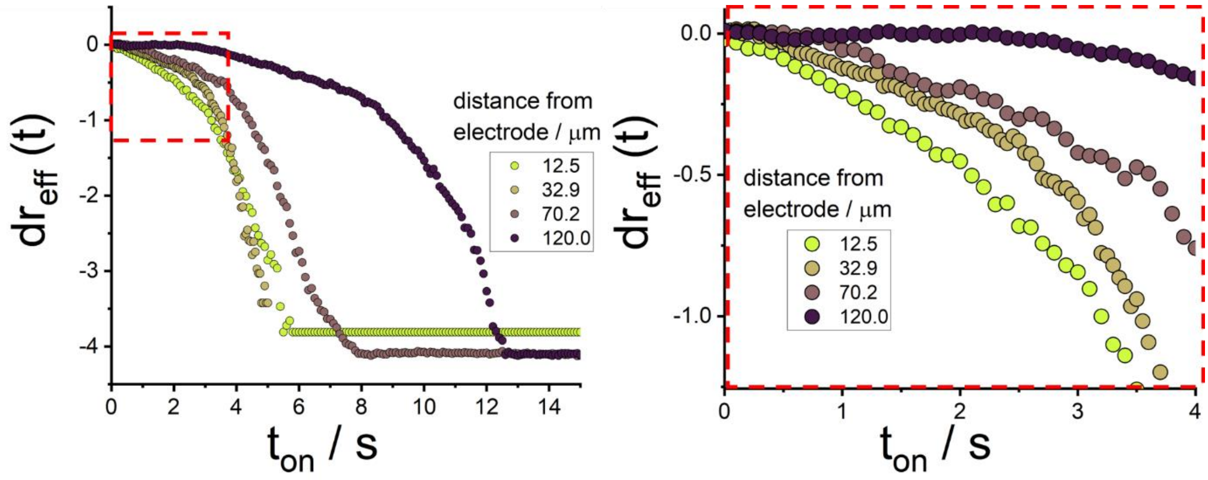

Figure S5. A plot of the change in the effective radius (dr_{eff}) of C.brauudii coccoliths during the opto-electrochemical experiments in 0.1M KNO_3 electrolyte. The distance of the coccolith from the electrode is shown in the legend. The y-axis plots the change in relative to that at t_{on} = 0, allowing the gradient to be directly compared for intra-species variation in coccolith size.

Section 5 Mass-transport of electrochemically generated reagents: numerical simulation

The opto-electrochemical cell (depicted in Figure S2) consists of a carbon fibre working electrode (r_e = 3.5 μm) in a cell volume of approximately 1 cm x 1 cm x 100 μm. In previous work the authors' have shown that the concentration profile radial from the working electrode in the opto-electrochemical cell can be simulated by a hemi-cylinder of equivalent radius on a surface.[8] To a good approximation and when within a distance less than or equal to the cell-height from the electrode, the diffusion field remains radial and Fick's second law in cylindrical coordinates can be used to describe the solution phase mass-transport.[8] In the absence of coupled homogeneous kinetics the concentration of species i, is described by:

$$\frac{\partial [i]}{\partial t} = D_i \left(\frac{\partial^2 [i]}{\partial r^2} + \frac{2}{r}\frac{\partial [i]}{\partial r} \right) \qquad Eq.\ S2$$

where t is time (s), r is the radial distance from the centre of the cylinder and D_i is the diffusion coefficient of the ith species ($m^2\ s^{-1}$). We are concerned with the diffusional flux of protons (and H_2BQ) to and from the cylindrical wire electrode. The initial conditions we simulate are

$$t = 0,\ r_e \leq r \leq r_{max},\quad [H_2BQ]_r = [H_2BQ]_{bulk}, [H^+]_r \ \&\ [BQ]_r = 0mM \qquad Eq.\ S3$$

where r_e is the radius of the cylindrical electrode (r_e = 3.5μm), r_{max} is a point at a semi-infinite distance away from the electrode such that the concentration is unperturbed during the course of the reaction. The bulk and initial 1,4-dihydroxybenzene concentration is set to $[H_2BQ]_{bulk}$ = 10mM as per the opto-electrochemical experiment.

At the start of the opto-electrochemical experiments, the applied potential at the working electrode is stepped from 0V to an over-potential of +1.2V (vs Ag wire) to drive the full two-electrons two-protons oxidation of $H_2BQ(aq)$ at the electrode-solution interface (more of which see SI section 3)

$$H_2BQ(aq) - 2e^- \rightarrow BQ(aq) + 2H^+(aq) \qquad Eq.\ S4$$

The electrochemical reaction occurs within an electron tunnelling distance from the working electrode, leading to a depletion of H_2BQ at the electrode-solution interface

$$t > 0,\ [H_2BQ]_{r_e} = 0 \qquad Eq.\ S5$$

Concurrently, two protons are formed at the electrode-solution interface for each of H_2BQ undergoing oxidation

$$t > 0\ \&\ r = r_e,\quad D_{H_2BQ}\frac{\partial [H_2BQ]}{\partial r} = -2D_{H^+}\frac{\partial [H^+]}{\partial r} \qquad Eq.\ S6$$

Note that the electrogenerated BQ is not known to affect the dissolution kinetics of calcium carbonate and is not included in the simulation. The mass-transport of material is described by Fick's second law of diffusion in cylindrical coordinates Eq. S2

$$t > 0 \ \& \ r_e < r < r_{max} \begin{cases} \frac{\partial[H_2BQ]}{\partial t} = D_{H_2BQ}\left(\frac{\partial^2[H_2BQ]}{\partial r^2} + \frac{1}{r}\frac{\partial[H_2BQ]}{\partial r}\right) \\ \frac{\partial[H^+]}{\partial t} = D_{H^+}\left(\frac{\partial^2[H^+]}{\partial r^2} + \frac{1}{r}\frac{\partial[H^+]}{\partial r}\right) \end{cases} \qquad Eq.\ S7$$

At the outer simulation boundary condition ($r = r_{max}$) the bulk concentration is not perturbed during the timescale of the simulation.

$$r = r_{max} \begin{cases} [H_2BQ]_{\,r_{max}} = [H_2BQ]_{bulk} \\ [H^+]_{\,r_{max}} = 0 \end{cases}$$

$$where\ r_{max} = r_e + 6\sqrt{D_{H^+}t_{max}} \qquad Eq.\ S8$$

The simulation is discretized using a finite difference method and solved back-implicitly. Numerical convergence, mass conservation and benchmark against known analytical expression is performed to ensure numerical accuracy.[9]

Figure S6 a) shows the predicted temporal (t) and spatial (r) evolution of proton concentration $[H^+](r,t)$ within the opto-electrochemical cell. Similarly, Figure S6 b) depicts the proton profile as a function of time during the experiment, at difference distances from the electrode. We have not considered the consumption of the protons by the calcium carbonate and further we assume that the solution is unbuffered; experimentally this is equivalent to the case where 0.7 M KNO_3 is used as the electrolyte. However, the simulation gives the concentration profile of the protons in the absence of being consumed at $CaCO_3$ particle. Importantly the mass-transport to/from a microcylinder is convergent and consequently in this geometry the flux tends to a *quasi*-steady-state (“quasi” here denotes that in the longer time limit ($t > r^2/D$) the flux to the cylinder is proportional to the inverse of log time, as opposed to truly time independent). As can be seen in Figure S6 b), within seconds of the opto-electrochemical experiment, the proton concentration at a distance r away from the electrode is above millimolar.

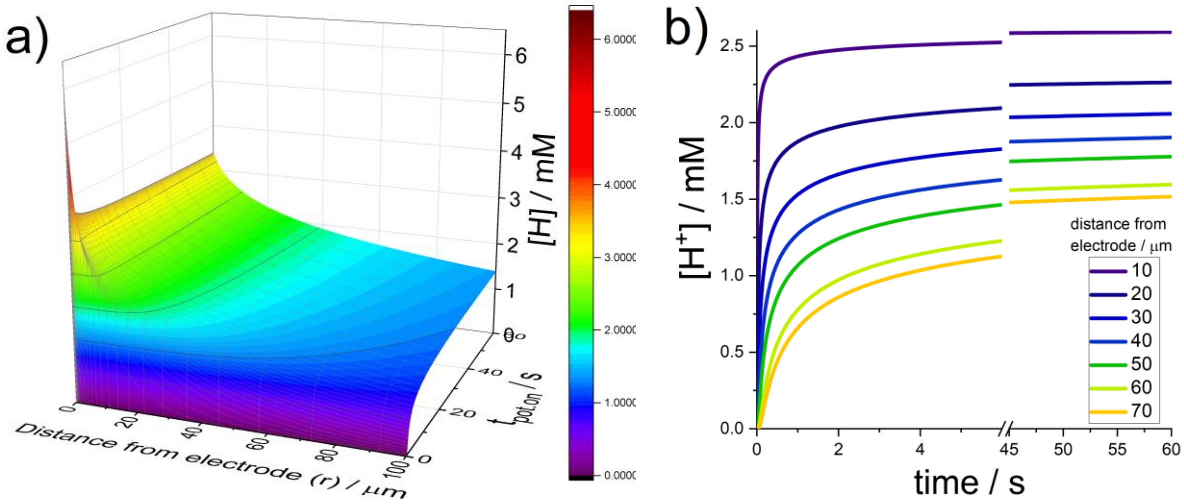

Figure S6. a) a plot of proton concentration profile as a function distance away from the cylindrical electrode (r_e = 3.5µm), at different time in the simulation. b) line plots of individual [H](t) profile at different distances from the cylindrical electrode. Other simulation parameters $[H_2BQ]_{bulk}$ = 10mM, D_{H2BQ} = 1.2 x 10^{-9} m^2s^{-1}, D_{H+} = 7.4 x $10^{-9}$$m^2s^{-1}$.

Section 6 Calcite particle dissolution kinetics

In this section, the acid dissolution of quasi-spherical-shaped calcite particles is first discussed. Upon exposure of the calcite to a homogeneous acidic chemical environment, the following chemical reaction occurs

$$CaCO_3(s) + H^+(aq) \overset{k_1}{\rightarrow} Ca^{2+}(aq) + HCO_3{}^-(aq) \qquad Eq.\ S9$$

where k_1 is the heterogeneous rate constant (cm s^{-1}). There are two important limits for this reaction. First, the reaction may be limited by the mass-transport of protons to the calcite interface. Second, the reaction may be limited by the surface reaction rate. As will be outlined below the switch in rate determining step is predominantly controlled by the size of the particle and the surface reaction rate. In previous work, using a channel-flow reactor and through electrochemically measuring the proton concentration downstream of a macro-sized Icelandic Spar (calcite) crystal a k_1 of 0.043(±0.015) cm s^{-1} has been measured. This value was obtained at pH < 4.0 in the inert electrolyte, KCl. Note that the term 'inert' means that neither K$^+$ nor Cl$^-$ are known to complex with the chemicals appearing in Eq. S9, thus the present of which is purely as electrolyte and does not affect the rate of calcite dissolution.

6.1 Surface reaction kinetics

The reaction flux at the particle-solution interface can be expressed as

$$D_{Ca^{2+}} \frac{\partial [Ca^{2+}]}{\partial r}\bigg|_{r=r_{particle}} = -D_{H^+} \frac{\partial [H^+]}{\partial r}\bigg|_{r=r_{particle}} = -k_1\, [H^+]_{surface} \quad Eq.\ S10$$

where r is radial distance from the centre of the spherical calcite particle, $r_{particle}$ is the radius of calcite particle, D_j is the diffusion coefficient of species j and $[j]$ is the concentration of species j. The subscript 'surface' denotes the surface concentration at the particle-solution interface.

For every mole of Ca^{2+}(aq) released into the solution, one mole of $CaCO_3(s)$ must have reacted with acid. Therefore, it is helpful to express Eq. S10 in terms of the rate in change of the particle size. First, the reaction flux (Eq. S10) is multiplied by the surface area of the calcite particle to obtain the rate of reaction

$$D_{Ca^{2+}} \frac{\partial [Ca^{2+}]}{\partial r}\bigg|_{r=r_{particle}} * Area = \frac{\partial (mols\ of\ CaCO_3(s))}{\partial t} = -\, k_1\, [H^+]_{surface} * Area \qquad Eq.\ S11$$

Since the number of moles of $CaCO_3(s)$ in a spherical calcite particle is equal to the volume multiplied by the density of calcite $\left(\rho_{CaCO_3} = 2.71\ x\ 10^{-4} mol\ m^{-3}\right)$, Eq. S11 becomes

$$\frac{\partial (mols\ of\ CaCO_3(s))}{\partial t} = \frac{\partial (V * \rho_{CaCO_3})}{\partial t} = -k_1\, [H^+]_{surface} 4\pi {r_{particle}}^2 \qquad Eq.\ S12$$

using chain rule, $\frac{\partial V}{\partial t}$ can be expressed in terms of $\frac{\partial r_{particle}}{\partial t}$

$$\frac{\partial V}{\partial t} = \frac{\partial V}{\partial r_{particle}} \frac{\partial r_{particle}}{\partial t} = 4\pi {r_{particle}}^2 \frac{\partial r_{particle}}{\partial t} \qquad Eq.\ S13$$

Substituting Eq. S13 into Eq. S12 and rearranging give

$$\frac{\partial r_{particle}}{\partial t} = -\frac{k_1}{\rho_{CaCO_3}}[H^+]_{surface} \qquad Eq.\ S14$$

Eq. S14 describes the rate of particle shrinkage for a perfectly smooth spherical calcite particle dissolving under a highly acid chemical environment (pH<4.0) with nothing else present in the solution other than inert electrolytes. In other words, it describes the rate in the absence of calcite dissolution inhibitors. Since k_1, and ρ_{CaCO_3} are constants, the rate of particle shrinkage is directly proportional to the proton concentration at the particle-solution interface. The higher the surface proton concentration, the quicker the calcite dissolves and shrink.

In opto-electrochemical experiments, the dissolution of coccoliths were not only conducted in inert electrolytes (0.7M KNO_3) but also in phytoplankton culture mediums. Note that KNO_3 is considered inert because neither K^+ (as above) nor NO_3^- are not known to complex with chemicals involved in the calcite dissolution reaction. The phytoplankton culture medium contains a mixture of electrolytes, minerals, nutrients and metal ion chelators, not too dissimilar to those found in natural seawater. Notably, this includes 54mM of Mg^{2+} (aq) and 2.4mM of Eq. S14 is generalized to

$$\frac{\partial r_{particle}}{\partial t} = -\frac{k_1\, c_p}{\rho_{CaCO_3}}[H^+]_{surface} \qquad Eq.\ S15$$

where an additional variable c_p has been added to account for the fact that the interfacial kinetics may deviate from the case of an idealized smooth spherical particle. Notably, the surface roughness of the calcite particle will serve to increase the rate (c_p >1), whereas as demonstrated by data shown in the main text and Table S3, the presence of a carbonate buffer and magnesium ions also decrease the reaction rate. Trace metal and organic carbon impurities within biogenic coccoliths may also affect c_p.

6.2 Mass-transport and the effect of particle size

The expression for the rate of dissolution given in Eq. S15 is proportional to the surface proton concentration. For an *isolated* particle we can consider how this surface proton concentration varies during the course of a reaction.

To approach this problem we simulate the surface proton concentration at a spherical calcite particle when exposed to a highly acidic solution. At t = 0, a spherical calcite particle is exposed to a homogeneous solution of proton $[H^+]_{bulk}$. The proton concentration is initially homogeneous throughout the solution

$$t = 0,\ r_{particle} \le r \le r_{max}:\quad [H^+]_r = [H^+]_{bulk} \qquad Eq.\ S16$$

At t > 0, acid dissolution reaction at the particle surface leads to a consumption of proton, at a rate described by Eq. S14, accompanied by a concomitant replenishment of protons, diffusing down the concentration gradient from the semi-infinite bulk solution as described by Fickian diffusion laws (Eq. S2). The boundary conditions at t > 0 are:

$$t > 0 \begin{cases} r = r_{particle}, & D_{H^+} \left.\frac{\partial [H^+]}{\partial r}\right|_{r=r_{particle}} = -k_1\ [H^+]_{surface} \\ r_{particle} < r < r_{max}, & \frac{\partial [H^+]}{\partial t} = D_{H^+}\left(\frac{\partial^2 [H^+]}{\partial r^2} + \frac{2}{r}\frac{\partial [H^+]}{\partial r}\right) \\ r = r_{max}, & [H^+]_{\ r_{max}} = [H^+]_{bulk} \end{cases} \qquad Eq.\ S17$$

where $[H^+]$ is the concentration of protons (mol m-3), D_{H^+} is the diffusion coefficient of H^+ (mol m-2), t is time (s), r is the distance from the centre of particle and r_{max} is, again, a distance sufficient far away from the particle interface such that the concentration at r_{max} is unperturbed from the bulk concentration during the simulation

$$r_{max} = r_{particle} + 6\sqrt{D_{H^+} t_{max}} \qquad Eq.\ S18$$

As in Section 5, the differential equations are discretized using finite difference methods and solved backwards implicitly.[11] The simulation was benchmarked thoroughly with analytical expressions including tests such as conservation of mass and convergence study, more of which is detailed in authors' previous work.[9]

Figure S7 shows the $[H^+]_{surface}$ as a function of time for 'smooth' spherical calcite particles with different radius. As can be seen, a steady-state $[H^+]_{surface}$ is reached within a couple of seconds of simulation as a result of the high diffusion coefficient of H^+ in aqueous solution. As the size of particle increases from sub-micron to larger than 10 microns, the $[H^+]_{surface}$ at steady-state is seen to decrease to a value less than 60% of the bulk proton concentration; this is a classic change in the convergent diffusion regime for when the particle size is small compare to the diffusion layer thickness, to that of a 'linear' diffusion regime when the particle size is big compare to the diffusion layer thickness.

Figure S8 plots the steady-state $[H^+]_{surface}$ for particle with different surface roughness, as expressed by increasing the value of c_p in equation Eq. S15. As the surface of the particle becomes rougher (high c_p), more surface-area of calcite is available to react, thus leading to a decrease in the surface proton concentration.

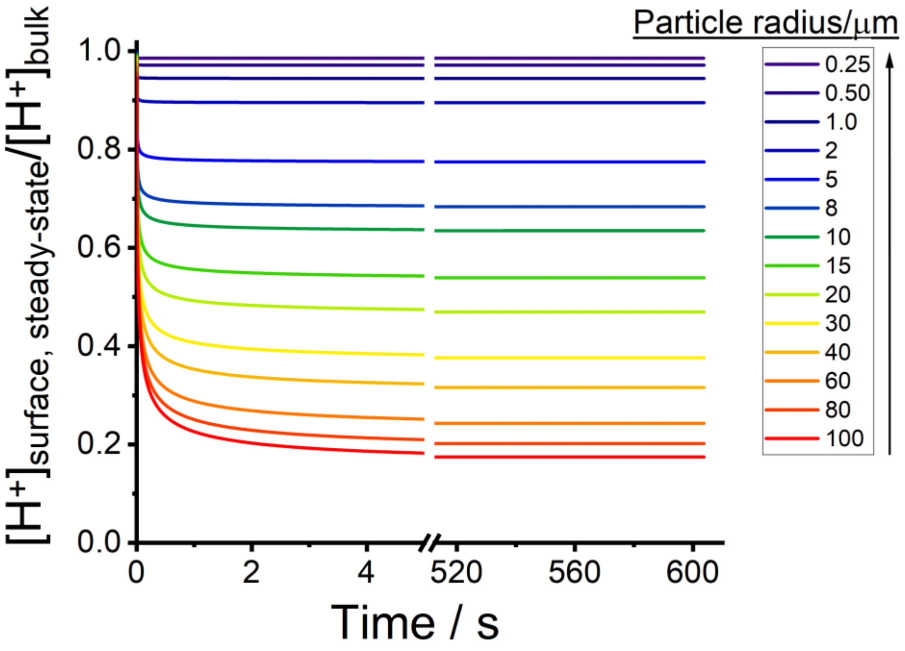

Figure S7. A plot of surface proton concentration with time, normalized to the bulk concentration of protons. Other simulation parameters: $k_1 = 0.043$ *cm* s^{-1}, $D_{H+} = 7.4x10^{-9} m^2 s^{-1}$, $c_p = 1$ *and the range of* $r_{particle}$ *is shown in graph legend (0.25 – 100 μm).*

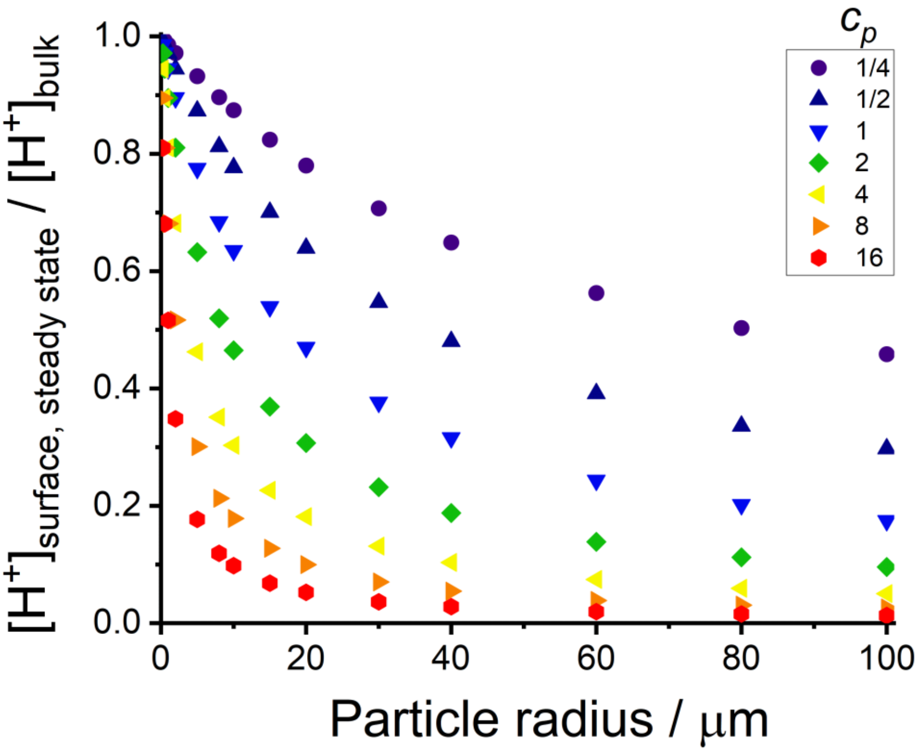

Figure S8. A plot of the steady-state surface proton concentration, relative to the bulk concentration, as a function of both the radius of the particle and c_p *values. Other simulation parameters:* $k_1 = 0.043$ *cm* s^{-1}, $D_{H+} = 7.4 \times 10^{-9} m^2 s^{-1}$ *and the range of* $r_{particle}$ *and* c_p *is shown in the plot.*

In the main text of this study, the dissolution of coccoliths is analysed as of disk-like calcite particles with a non-unity surface roughness factor. The largest of all species in this study (C.*braaudii*) has a typical coccolith thickness

of 1-2 µm and r_{eff} < 6 µm. It can be inferred from Figure S8 that if a calcite particle, with equivalent dimensions to that of a coccolith, were to be exposed to a strong acidic chemical environment, the deviation in $[H^+]_{surface}$ is no more than ~30% from $[H^+]_{bulk}$. For the smaller types of coccoliths, such as those found bearing on E.*huxleyi* (typically measured thickness < 0.7 µm and r_{eff} < 2.5µm) the $[H^+]_{surface}$ is approximately equal to $[H^+]_{bulk}$. Therefore, the dissolution of coccoliths in a strong acidic environment occurs within a regime controlled by the surface reaction rate $\left([H^+]_{surface} \approx [H^+]_{bulk}\right)$, and not the mass-transport of protons to the particle $\left([H^+]_{surface} \approx 0\right)$.

6.3 Experimental verification of the dissolution kinetics

In order to verify the above model, the dissolution of a solid calcite particle was studied in the electrochemical cell. Figure S9 shows the rate of calcite particle shrinkage (r_{eff} = 5.4 µm) when exposed to the strong acidic environment, the experimental conditions mirror those used in the coccolith in the opto-electrochemical experiment. The purple squares and red dots represents two approaches in quantifying the particle size from image analysis. The green dashed line is the expected dissolution rate for a solid particle as numerically predicted based on the above model and where c_p =1. The initial slope (µm s^{-1}) within the first tens of seconds matches with excellent agreement to that numerically predicted rate of shrinkage when $[H^+]_{surface} = [H^+](r,t)$; where $[H^+](r,t)$ is the numerically simulated proton concentration in the opto-electrochemical experiment – see SI section 5. Notice the initial slope deviates from linearity after 20 seconds is similar to the 'dog leg' kinetics seen with the dissolution of coccoliths. This is due to the start of the dissolution of the calcite particle perpendicular to the projection plane of the 2D image.

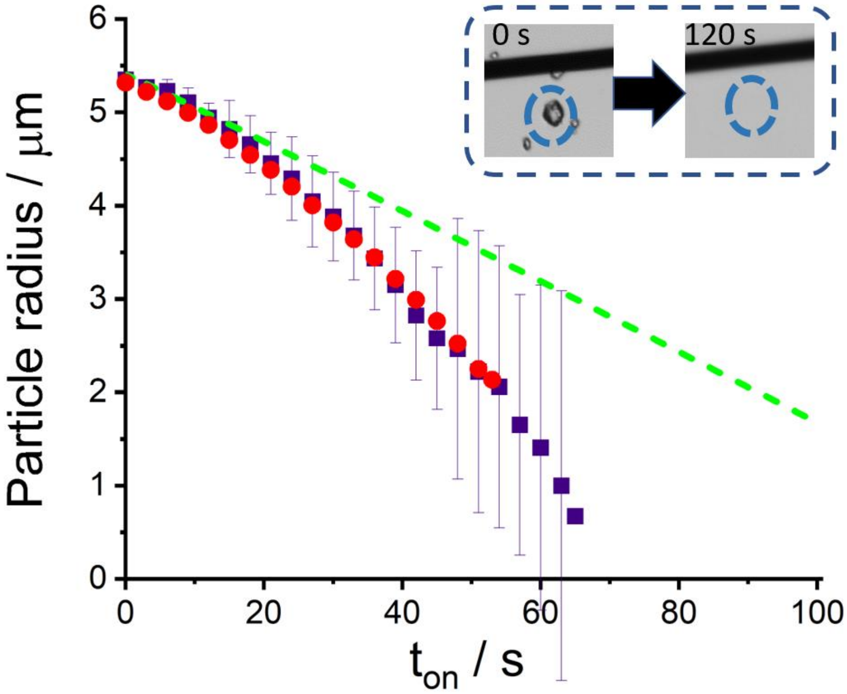

Figure S9. The change in the particle radius of a quasi-spherical detrital calcite particle with time(s) at a distance of 16µm from the carbon-fibre wire electrode (r_e = 3.5µm). At the start of the experiment (t_{on} = 0s), the potential applied to the wire electrode is step from 0V to +1.2V (vs Ag wire) to fully drive the oxidation of 10mM $H_2BQ(aq)$ in the 0.7M KNO_3 electrolyte. The dissolution of the detrital particle analysis via two approaches: purple square – the particle radius measured from the centre of mass (average with error bar) and the red dots is the r_{eff} calculated from the projection area after image threshold, where πr_{eff}^2=area. Overlaid in green dotted line is the predicted dissolution rate occurs at surface-area controlled, more of which see below. Inlay shows the detrital particle at t_{on} = 0s and at t_{on} = 120s.

6.4 Numerically predicted dissolution kinetics of a calcite particle as a function of distance from the electrode

Figure S10 predicts the shrinkage of perfectly spherical solid calcite particles in the opto-electrochemical experiment. The change in particle-size as a function of time is calculated via Eq. S15, using the proton concentration profile $[H^+](r,t)$ shown in Figure S6 – simulated for an inert electrolyte ($c_p = 1$). The initial dissolution rate, $(dr_{particle}/dt)$ in Figure S10, is shown to be sensitive to the distance of particle from the electrode, with a faster dissolution rate for particle near to the electrode. A particle distance of 70 µm from the electrode, however, the dissolution rate at t ≈ 0s is sluggish due to the finite time required for the proton to diffusion from the electrode, but over the course of ~ 2-3s, the slope is approximately averaged to a constant value. This is fully consistent with that seen experimental for dissolution of coccolith, as shown in Figure S5.

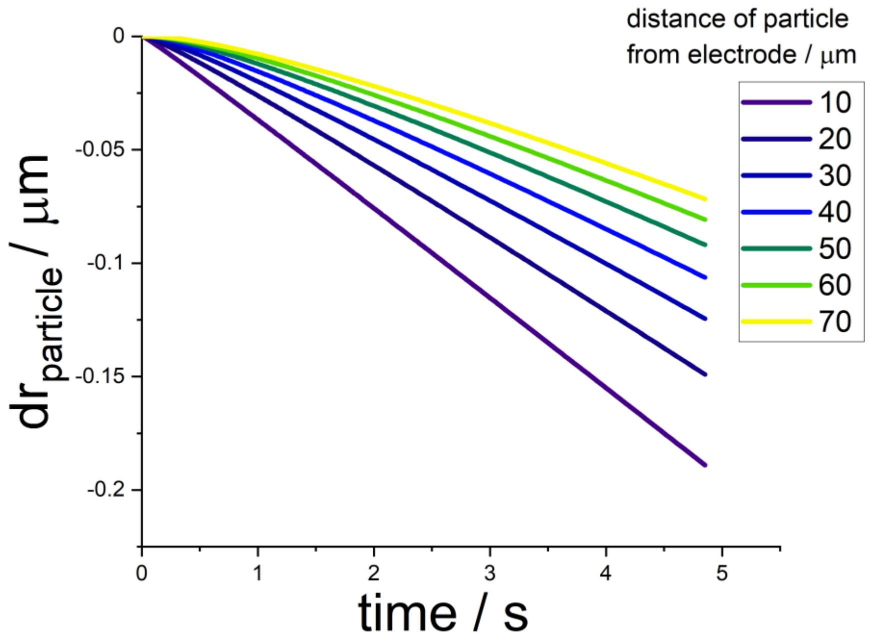

Figure S10. Shrinkage of solid calcite particles ($c_p = 1$) as a function of time in the opto-electrochemical cell. The particle distance from the electrode (r) is shown in the legend. The proton concentration $[H](r,t)$ used for the calculation is reported in Figure S6.

Section 7 Coccolith data

The table below tabulates all coccolith data shown in the main text.

Table S3. Tabulated Coccolith data from opto-electrochemical experiments. Distance from electrode was measured from the electrode edge to coccolith centre. All the electrolyte has an addition of 10mM H_2BQ acid precursor prior to the experiment. The concentration of the chemical components are: 0.7M KNO_3, 54.6mM Mg^{2+} and 2.4mM HCO_3^-. See SI Section 1.2 for K/2 culture medium. Effective radius (r_{eff}) was calculated from the coccoliths projection area measured after image threshold (Area = πr_{eff}^2). Coccolith length denotes the longest length of the coccolith, which is equivalent to the major axis for elliptical liths. The initial slope was the initial dissolution rate dr_{eff}/dt, obtained from t_{on} = 0s as shown in Figure S5. Maximum thickness was obtained from the coccolith volume reconstruction. c_p was the factor increase in dissolution rate as compared to a smooth and solid calcite particle with radius r_{eff}. Coccolith volume was obtained from image reconstruction and coccolith mass was estimated using a calcite density of 2.71 g cm^{-3}.

Species	Electrolyte	Distance from electrode / µm	r_{eff} / µm	Coccolith length / µm	Initial slope / µm s^{-1}	Maximum thickness / µm	c_p	Coccolith volume / µm³	Coccolith mass / pg
**C.braaudii**	KNO_3	13.5	4.1	8.6	0.16	0.9	4.4	31.0	82.3
**C.braaudii**	KNO_3	32.9	3.4	7.6	0.16	0.8	6.3	21.0	53.9
**C.braaudii**	KNO_3	33.2	2.9	7.0	0.12	0.5	4.8	15.0	40.7
**C.braaudii**	KNO_3	32.8	3.9	7.9	0.11	0.8	4.1	32.0	53.4
**C.braaudii**	KNO_3	42.8	5.1	10.8	0.06	1.2	5.5	105.0	159.9
**C.braaudii**	KNO_3	61.2	3.8	7.7	0.06	0.4	5.5	26.6	21.4
**C.braaudii**	KNO_3	79.0	4.4	8.8	0.13	0.9	5.7	42.5	115.2
**C.braaudii**	KNO_3	42.4	5.0	10.6	0.16	1.4	8.2	72.8	197.3
**C.braaudii**	KNO_3	54.3	4.3	9.9	0.10	0.7	7.1	27.2	73.7
**C.braaudii**	KNO_3	70.2	4.8	10.2	0.13	1.0	12	42.5	115.2
**C.braaudii**	KNO_3	40.8	4.5	10.2	0.10	0.9	5.4	38.4	104.1
**C.braaudii**	KNO_3	21.2	4.6	10.0	0.13	1.1	4.5	45.6	123.6
**C.braaudii**	KNO_3	34.5	4.0	9.2	0.19	1.1	8.7	34.6	93.8
**C.braaudii**	KNO_3	11.4	4.7	10.2	0.20	2.0	5.4	72.6	196.7
**C.braaudii**	KNO_3	27.1	4.6	10.6	0.087	1.1	3.6	50.3	136.3
**C.braaudii**	KNO_3	27.7	5.1	11.3	0.12	2.1	5.1	98.9	268.0
**C.braaudii**	KNO_3	29.2	4.5	9.8	0.082	1.2	3.5	50.2	136.0
**C.braaudii**	KNO_3	12.5	3.8	8.3	0.24	1.3	7.0	36.3	98.4
**C.braaudii**	KNO_3	21.6	4.8	11.5	0.27	1.4	9.5	68.5	185.6
**C.braaudii**	KNO_3	10.1	5.1	9.3	0.47	2.1	12	62.8	170.2
**C.braaudii**	KNO_3	40.3	4.3	9.4	0.16	1.0	7.9	37.9	102.7
**C.braaudii**	$KNO_3 + Mg^{2+}$	23.1	3.3	7.6	0.15	0.5	5.1	8.5	23.1
**C.braaudii**	$KNO_3 + Mg^{2+}$	35.2	4.2	9.3	0.094	0.6	4.0	20.0	54.3
**C.braaudii**	$KNO_3 + Mg^{2+}$	86.3	5.3	10.8	0.10	1.3	8.4	73.1	198.1
**C.braaudii**	$KNO_3 + Mg^{2+}$	15.3	3.6	8.0	0.17	0.9	5.1	17.3	46.9
**C.braaudii**	$KNO_3 + Mg^{2+}$	22.2	4.7	10.4	0.20	1.8	6.9	82.7	224.1
**C.braaudii**	$KNO_3 + Mg^{2+}$	23.2	4.9	10.4	0.11	1.1	3.7	54.7	148.2
**C.braaudii**	$KNO_3 + Mg^{2+}$	34.7	4.0	9.7	0.15	1.2	6.1	34.5	93.5
**C.braaudii**	$KNO_3 + Mg^{2+}$	38.5	3.9	9.1	0.13	1.2	5.6	39.5	107.0
**C.braaudii**	$KNO_3 + Mg^{2+}$	50.7	4.5	9.6	0.14	1.0	7.2	34.4	93.2
**C.braaudii**	$KNO_3 + Mg^{2+}$	57.3	4.7	9.9	0.072	1.0	4.2	44.2	119.8
**C.braaudii**	$KNO_3 + Mg^{2+}$	70.2	4.8	10.6	0.053	0.7	3.6	34.1	92.4
**C.braaudii**	$KNO_3 + Mg^{2+}$	17.5	4.9	10.2	0.042	0.6	1.3	24.9	67.5
**C.braaudii**	$KNO_3 + Mg^{2+}$	27.2	4.4	9.9	0.046	0.6	1.7	29.0	78.5
**C.braaudii**	$KNO_3 + Mg^{2+}$	24.9	4.9	10.8	0.063	1.0	2.3	45.6	123.6
**C.braaudii**	$KNO_3 + Mg^{2+}$	26.7	3.9	9.2	0.088	0.9	3.2	28.4	77.0
**C.braaudii**	$KNO_3 + Mg^{2+}$	28.6	5.0	11.4	0.10	1.3	3.9	66.3	179.7
**C.braaudii**	$KNO_3 + Mg^{2+}$	44.4	4.1	7.9	0.085	0.8	4.2	29.4	79.7

SUPPORTING INFORMATION

Species	Electrolyte	Distance from electrode / µm	r_{eff} / µm	Coccolith length / µm	Initial slope / µm s^{-1}	Maximum thickness / µm	c_p	Coccolith volume / µm³	Coccolith mass / pg
C.braaudii	$KNO_3 + Mg^{2+}$	65.6	4.5	9.9	0.053	0.7	3.4	28.8	78.0
C.braaudii	$KNO_3 + Mg^{2+}$	48.3	3.6	8.3	0.085	0.7	4.4	22.6	61.2
C.braaudii	$KNO_3 + Mg^{2+}$	51.2	3.4	8.0	0.099	0.7	5.3	21.2	57.5
C.braaudii	$KNO_3 + Mg^{2+}$	27.6	4.7	10.5	0.14	1.2	5.4	56.0	151.7
C.braaudii	$KNO_3 + Mg^{2+}$	29.6	3.7	8.3	0.14	1.0	5.3	27.5	74.5
C.braaudii	$KNO_3 + Mg^{2+}$	64.1	3.8	8.9	0.072	0.6	4.6	18.6	50.4
C.braaudii	$KNO_3 + HCO_3^-$	34.7	5.3	11.4	0.13	2.1	5.5	110.5	299.5
C.braaudii	$KNO_3 + HCO_3^-$	86.3	4.3	9.4	0.054	1.1	4.4	40.0	108.4
C.braaudii	$KNO_3 + HCO_3^-$	86.9	5.1	11.0	0.055	1.2	4.5	64.4	174.5
C.braaudii	$KNO_3 + HCO_3^-$	16.5	3.9	9.4	0.13	1.1	3.9	37.4	101.3
C.braaudii	$KNO_3 + HCO_3^-$	56.1	4.5	10.5	0.045	1.0	2.6	38.0	103.0
C.braaudii	$KNO_3 + HCO_3^-$	22.5	4.8	10.8	0.13	1.7	4.6	71.7	194.3
C.braaudii	$KNO_3 + HCO_3^-$	59.8	4.2	9.5	0.032	0.6	1.9	25.1	68.0
C.braaudii	$KNO_3 + HCO_3^-$	18.5	4.3	9.4	0.16	2.3	5.0	64.9	175.9
C.braaudii	$KNO_3 + HCO_3^-$	7.6	4.5	9.8	0.12	1.4	2.9	51.5	139.6
C.braaudii	$KNO_3 + HCO_3^-$	5.8	3.8	8.5	0.15	1.3	3.5	27.9	75.5
C.braaudii	$KNO_3 + HCO_3^-$	6.5	3.8	7.9	0.11	1.1	2.5	27.7	75.1
C.braaudii	$KNO_3 + HCO_3^-$	10.6	4.1	8.5	0.043	0.9	1.1	30.5	82.6
C.braaudii	$KNO_3 + HCO_3^-$	20.4	4.6	8.8	0.031	0.6	1.0	26.1	70.6
C.braaudii	$KNO_3 + HCO_3^-$	11.4	4.7	10.8	0.15	1.5	4.1	58.1	157.4
C.braaudii	$KNO_3 + HCO_3^-$	19.5	3.8	8.1	0.18	1.0	5.9	24.0	65.1
C.braaudii	$KNO_3 + HCO_3^-$	23.9	4.4	9.3	0.098	0.9	3.5	35.9	97.2
C.braaudii	$KNO_3 + HCO_3^-$	23.9	4.9	10.2	0.13	1.1	4.5	48.8	132.3
C.braaudii	$KNO_3 + HCO_3^-$	47.2	4.1	8.6	0.091	0.8	4.6	31.9	86.4
C.braaudii	$KNO_3 + HCO_3^-$	9.4	3.9	8.3	0.29	2.1	7.3	71.7	194.4
C.braaudii	$KNO_3 + HCO_3^-$	9.7	3.9	9.4	0.080	0.9	2.0	32.8	88.9
C.braaudii	K/2	41.9	5.0	10.9	0.12	1.7	5.8	90.8	246.0
C.braaudii	K/2	10.6	4.7	10.7	0.076	1.9	2.0	77.9	211.2
C.braaudii	K/2	26.8	4.8	10.1	0.070	1.5	2.6	68.9	186.6
C.braaudii	K/2	33.5	5.0	10.5	0.059	1.7	2.5	77.8	210.8
C.braaudii	K/2	61.8	3.9	9.4	0.037	0.8	2.3	25.2	68.2
C.braaudii	K/2	88.6	4.6	9.9	0.041	0.7	3.7	32.8	88.9
C.braaudii	K/2	16.7	4.5	9.3	0.067	1.0	2.0	38.4	104.0
C.braaudii	K/2	22.4	4.2	9.3	0.089	1.0	3.1	38.8	105.1
C.braaudii	K/2	25.0	3.4	7.3	0.095	0.9	3.4	20.4	55.4
C.braaudii	K/2	21.3	5.2	11.5	0.077	2.8	2.6	123.7	335.1
C.braaudii	K/2	28.0	5.0	10.7	0.063	2.1	2.4	90.0	243.8
C.braaudii	K/2	40.3	3.9	8.6	0.050	1.6	2.3	48.2	130.5
C.braaudii	K/2	45.5	4.8	10.6	0.054	1.4	2.7	73.8	199.9
C.braaudii	K/2	87.1	5.0	10.6	0.052	1.0	4.3	45.3	122.8
C.braaudii	K/2	19.3	4.2	9.9	0.092	1.1	3.0	40.6	110.1
C.braaudii	K/2	21.8	3.9	8.6	0.064	0.7	2.1	22.2	60.1
C.braaudii	K/2	20.1	4.0	8.4	0.098	1.0	3.2	45.3	122.8
E.huxleyi	K/2	11.2	1.1	2.3	0.33	0.3	8.9	0.7	1.8
E.huxleyi	K/2	20.5	1.4	3.4	0.097	0.2	3.2	1.1	2.9
E.huxleyi	K/2	21.0	1.6	3.7	0.34	0.6	11	2.8	7.6
E.huxleyi	K/2	21.5	2.2	4.7	0.11	0.4	3.9	4.8	13.1
E.huxleyi	K/2	24.6	1.6	3.6	0.21	0.8	7.6	3.1	8.4

SUPPORTING INFORMATION

Species	Electrolyte	Distance from electrode / µm	r_{eff} / µm	Coccolith length / µm	Initial slope / µm s^{-1}	Maximum thickness / µm	c_p	Coccolith volume / µm^3	Coccolith mass / pg
E.*huxleyi*	K/2	30.2	1.2	2.5	0.18	0.4	7.0	1.1	3.0
E.*huxleyi*	K/2	35.3	1.5	3.4	0.13	0.4	5.4	1.9	5.2
E.*huxleyi*	K/2	37.8	1.3	2.8	0.10	0.3	4.6	1.1	3.0
E.*huxleyi*	K/2	9.3	1.8	3.9	0.34	0.4	8.8	2.9	7.9
E.*huxleyi*	K/2	13.2	1.5	3.5	0.48	0.6	13	2.6	7.1
E.*huxleyi*	K/2	16.1	1.9	4.3	0.21	0.4	6.2	3.2	8.7
E.*huxleyi*	K/2	34.8	1.4	3.7	0.31	0.8	13	3.1	8.3
E.*huxleyi*	K/2	40.0	1.6	3.6	0.13	0.4	6.1	2.3	6.2
E.*huxleyi*	K/2	18.0	1.7	4.1	0.60	0.9	19	4.8	12.9
E.*huxleyi*	K/2	39.5	1.8	4.2	0.19	0.3	8.8	1.5	4.2
E.*huxleyi*	K/2	43.5	2.1	5.0	0.22	0.8	11	7.6	20.5
E.*huxleyi*	K/2	23.9	2.2	4.8	0.23	0.6	8.2	6.4	17.3
E.*huxleyi*	K/2	27.9	2.2	5.2	0.22	0.6	8.1	6.7	18.2
E.*huxleyi*	K/2	11.5	1.5	3.7	0.34	0.7	9.2	3.1	8.3
E.*huxleyi*	K/2	29.2	1.7	4.1	0.28	0.7	11	3.9	10.5
E.*huxleyi*	K/2	15.1	1.8	4.2	0.26	0.5	7.7	3.5	9.6
E.*huxleyi*	K/2	18.3	2.3	4.8	0.35	1.1	11	10.8	29.2
E.*huxleyi*	K/2	24.7	2.2	4.5	0.25	0.9	9.1	8.6	23.3
E.*huxleyi*	K/2	34.0	2.0	4.6	0.15	0.5	6.3	4.8	13.0
E.*huxleyi*	K/2	46.5	2.1	4.9	0.077	0.4	3.9	4.5	12.2
E.*huxleyi*	K/2	9.7	1.9	4.3	0.64	0.9	16	6.7	18.1
E.*huxleyi*	K/2	10.4	1.6	4.0	0.12	0.3	3.2	1.6	4.3
E.*huxleyi*	K/2	16.2	1.6	3.8	0.23	0.5	7.0	2.6	7.1
E.*huxleyi*	K/2	23.7	1.7	4.4	0.29	0.6	10	3.6	9.8
E.*huxleyi*	K/2	30.1	2.1	4.8	0.14	0.4	5.4	4.2	11.3
E.*huxleyi*	K/2	38.8	1.5	3.3	0.11	0.3	4.8	1.5	4.2
C.*leptoporus*	K/2	14.5	3.5	6.4	0.024	0.3	0.69	8.8	23.8
C.*leptoporus*	K/2	80.8	2.6	5.8	0.020	0.3	1.5	6.5	17.5
C.*leptoporus*	K/2	81.2	2.7	5.8	0.023	0.5	1.8	6.2	16.8
C.*leptoporus*	K/2	76.6	2.9	6.4	0.012	0.2	0.88	4.4	11.9
C.*leptoporus*	K/2	24.0	1.8	4.2	0.095	0.5	3.4	3.3	8.9
C.*leptoporus*	K/2	94.8	2.9	6.4	0.010	0.2	0.93	4.8	12.9
C.*leptoporus*	K/2	29.7	2.2	4.5	0.056	0.5	2.1	4.8	13.0
C.*leptoporus*	K/2	54.0	2.0	4.6	0.027	0.4	1.5	7.8	21.1
C.*leptoporus*	K/2	44.2	3.3	7.2	0.034	0.6	1.6	14.5	39.2
C.*leptoporus*	K/2	55.4	1.9	4.1	0.13	0.9	7.4	6.1	16.4
C.*leptoporus*	K/2	46.5	3.1	6.4	0.019	0.4	0.96	7.8	21.1
C.*leptoporus*	K/2	53.8	3.2	6.5	0.027	0.6	1.5	11.8	31.9
C.*leptoporus*	K/2	69.7	3.4	7.0	0.009	0.3	0.60	6.7	18.2
C.*leptoporus*	K/2	30.2	2.1	5.0	0.074	0.5	2.9	4.4	12.0
C.*leptoporus*	K/2	38.1	3.1	6.2	0.045	0.5	2.0	11.0	29.7
C.*leptoporus*	K/2	40.7	3.2	6.2	0.088	1.0	4.1	19.6	53.2
C.*leptoporus*	K/2	19.3	3.4	7.2	0.029	0.3	0.93	8.7	23.6
C.*leptoporus*	K/2	31.5	3.7	7.2	0.043	0.6	1.7	18.5	50.1
C.*leptoporus*	K/2	53.7	2.4	4.8	0.029	0.3	1.6	4.0	11.0
C.*leptoporus*	K/2	56.2	3.2	6.8	0.028	0.4	1.6	9.1	24.6
C.*leptoporus*	K/2	59.3	3.2	6.6	0.032	0.5	1.9	11.8	32.0

Species	Electrolyte	Distance from electrode / µm	r_{eff} / µm	Coccolith length / µm	Initial slope / µm s^{-1}	Maximum thickness / µm	c_p	Coccolith volume / µm^3	Coccolith mass / pg
**G.*oceanica***	K/2	16.6	2.2	4.7	0.14	0.5	4.2	5.5	14.9
**G.*oceanica***	K/2	33.4	1.9	4.7	0.10	0.7	4.3	3.3	9.0
**G.*oceanica***	K/2	37.0	2.4	5.7	0.19	0.9	8.1	8.4	22.7
**G.*oceanica***	K/2	8.6	2.6	5.7	0.12	0.9	2.9	13.7	37.2
**G.*oceanica***	K/2	14.1	2.0	5.1	0.22	1.2	6.2	10.6	28.6
**G.*oceanica***	K/2	16.9	2.4	4.9	0.25	1.3	7.5	14.8	40.0
**G.*oceanica***	K/2	15.3	1.8	4.3	0.21	0.9	6.3	8.4	22.8
**G.*oceanica***	K/2	36.1	1.8	3.9	0.16	1.1	7.1	7.8	21.1
**G.*oceanica***	K/2	44.1	1.5	3.7	0.093	0.7	4.5	4.5	12.1
**G.*oceanica***	K/2	54.0	3.2	7.6	0.044	0.6	2.4	15.2	41.2
**G.*oceanica***	K/2	22.0	2.7	6.1	0.060	0.5	2.0	8.6	23.3
**G.*oceanica***	K/2	29.3	3.1	6.3	0.087	0.9	3.4	19.4	52.6
**G.*oceanica***	K/2	52.9	2.5	5.6	0.082	0.8	4.5	15.8	42.7
**G.*oceanica***	K/2	11.7	2.1	4.8	0.14	0.9	3.7	8.8	23.8
**G.*oceanica***	K/2	16.4	2.1	5.0	0.18	1.1	5.4	11.5	31.0
**G.*oceanica***	K/2	18.2	2.0	4.1	0.14	1.2	4.4	11.0	29.9
**G.*oceanica***	K/2	20.5	1.9	4.5	0.067	0.5	2.2	4.4	11.8
**G.*oceanica***	K/2	47.4	2.6	5.7	0.11	1.0	5.8	16.2	44.0
**G.*oceanica***	K/2	11.8	3.5	7.3	0.12	0.9	3.3	23.7	64.3
**G.*oceanica***	K/2	25.6	3.4	7.7	0.10	1.0	3.7	24.0	65.1
**G.*oceanica***	K/2	13.7	3.0	6.6	0.20	1.5	5.6	27.5	74.5
**G.*oceanica***	K/2	34.1	2.9	6.4	0.087	0.9	3.6	16.7	45.3
**G.*oceanica***	K/2	38.7	2.7	5.9	0.13	1.1	5.9	13.9	37.8
**G.*oceanica***	K/2	24.5	2.4	5.3	0.27	1.7	9.6	22.2	60.2
**G.*oceanica***	K/2	33.8	2.2	5.2	0.23	1.4	9.5	14.7	39.8
**G.*oceanica***	K/2	49.8	2.7	6.0	0.11	0.9	5.6	14.9	40.4
**G.*oceanica***	K/2	50.7	3.4	7.4	0.097	1.0	5.1	23.1	62.6

References

1. M. D. Keller, R. C. Selvin, W. Claus and R. R. Guillard, *Journal of Phycology*, 1987, **23**, 633-638.
2. F. M. Morel, J. Rueter, D. M. Anderson and R. Guillard, *Journal of Phycology*, 1979, **15**, 135-141.
3. C. Batchelor-McAuley, J. Ellison, K. Tschulik, P. L. Hurst, R. Boldt and R. G. Compton, *Analyst*, 2015, **140**, 5048-5054.
4. J. Schindelin, I. Arganda-Carreras, E. Frise, V. Kaynig, M. Longair, T. Pietzsch, S. Preibisch, C. Rueden, S. Saalfeld and B. Schmid, *Nature Methods*, 2012, **9**, 676-682.
5. J. W. Anthony, *Handbook of Mineralogy: Borates, carbonates, sulfates*, Mineral Data Pub., 1990.
6. M. Quan, D. Sanchez, M. F. Wasylkiw and D. K. Smith, *Journal of the American Chemical Society*, 2007, **129**, 12847-12856.
7. M. Yang, C. Batchelor-McAuley, E. Kätelhön and R. G. Compton, *Analytical Chemistry*, 2017, **89**, 6870-6877.
8. J. Ma, M. Yang, C. Batchelor-McAuley and R. G. Compton, *Physical Chemistry Chemical Physics*, 2020, **22**, 12422-12433.
9. E. Kätelhön and R. G. Compton, *Analyst*, 2015, **140**, 2592-2598.
10. R. G. Compton and C. A. Brown, *Journal of Colloid and Interface Science*, 1994, **165**, 445-449.

11. R. G. Compton, E. Katelhon, K. R. Ward and E. Laborda, *Understanding Voltammetry: Simulation of Electrode Processes*, World Scientific, 2020.

Author Contributions

Conceptualization: MY, CBM, RGC, REMR, HAB
Methodology: MY, CBM
Experiment: MY (lead), SB, CBM
Formal analysis: MY
Visualization: MY
Supervision: RGC
Writing—original draft: MY (equal), CBM (equal), RGC
Writing—review & editing: MY, CBM, RGC, REMR, HAB
